# Wild-type sTREM2 blocks Aβ aggregation and neurotoxicity, but the Alzheimer's R47H mutant increases Aβ aggregation

**DOI:** 10.1016/j.jbc.2021.100631

**Published:** 2021-04-03

**Authors:** Anna Vilalta, Ye Zhou, Jean Sevalle, Jennifer K. Griffin, Kanayo Satoh, David H. Allendorf, Suman De, Mar Puigdellívol, Arturas Bruzas, Miguel A. Burguillos, Roger B. Dodd, Fusheng Chen, Yalun Zhang, Patrick Flagmeier, Lisa-Maria Needham, Masahiro Enomoto, Seema Qamar, James Henderson, Jochen Walter, Paul E. Fraser, David Klenerman, Steven F. Lee, Peter St George-Hyslop, Guy C. Brown

**Affiliations:** 1 Department of Biochemistry, University of Cambridge, Cambridge, United Kingdom; 2 Departments of Medicine (Neurology) and Medical Biophysics, University of Toronto and University Health Network, Toronto, Ontario, Canada; 3 AstraZeneca, Cambridge, United Kingdom; 4 Cambridge Institute for Medical Research, Cambridge, United Kingdom; 5 Department of Chemistry, University of Cambridge, Cambridge, United Kingdom; 6 Princess Margaret Cancer Centre, University Health Network, Department of Medical Biophysics, University of Toronto, Toronto, Ontario, Canada; 7 Molecular Cell Biology, Department of Neurology, University of Bonn, Bonn, Germany; 8 Cambridge Dementia Research Institute, Cambridge Biomedical Campus, Cambridge, United Kingdom

**Keywords:** amyloid beta, Alzheimer's disease, TREM2, microglia, oligomers, neurotoxicity, sTREM2, AD, Alzheimer's disease, BLI, bio-layer interferometry, CSF, cerebrospinal fluid, CTF, C-terminal fragment, HPLC-SEC, high-performance liquid chromatography size-exclusion chromatography, MCI, mild cognitive impairment, TEM, transmission electron microscopy, TIRF, total internal reflection fluorescence, TREM2, triggering receptor expressed on myeloid cells 2

## Abstract

TREM2 is a pattern recognition receptor, expressed on microglia and myeloid cells, detecting lipids and Aβ and inducing an innate immune response. Missense mutations (*e.g.*, R47H) of TREM2 increase risk of Alzheimer's disease (AD). The soluble ectodomain of wild-type TREM2 (sTREM2) has been shown to protect against AD *in vivo*, but the underlying mechanisms are unclear. We show that Aβ oligomers bind to cellular TREM2, inducing shedding of the sTREM2 domain. Wild-type sTREM2 bound to Aβ oligomers (measured by single-molecule imaging, dot blots, and Bio-Layer Interferometry) inhibited Aβ oligomerization and disaggregated preformed Aβ oligomers and protofibrils (measured by transmission electron microscopy, dot blots, and size-exclusion chromatography). Wild-type sTREM2 also inhibited Aβ fibrillization (measured by imaging and thioflavin T fluorescence) and blocked Aβ-induced neurotoxicity (measured by permeabilization of artificial membranes and by loss of neurons in primary neuronal–glial cocultures). In contrast, the R47H AD-risk variant of sTREM2 is less able to bind and disaggregate oligomeric Aβ but rather promotes Aβ protofibril formation and neurotoxicity. Thus, in addition to inducing an immune response, wild-type TREM2 may protect against amyloid pathology by the Aβ-induced release of sTREM2, which blocks Aβ aggregation and neurotoxicity. In contrast, R47H sTREM2 promotes Aβ aggregation into protofibril that may be toxic to neurons. These findings may explain how wild-type sTREM2 apparently protects against AD *in vivo* and why a single copy of the R47H variant gene is associated with increased AD risk.

A prominent neuropathological feature of Alzheimer's disease (AD) is the presence of extracellular deposits of the amyloid β-peptide in amyloid plaques, surrounded by activated microglia (1, 2, 3). The importance of this microglial response to the pathogenesis of AD has been highlighted by the recent discovery of sequence variants in multiple genes expressed in microglia that alter risk for AD. Prominent among these microglial AD-risk genes is the “triggering receptor expressed on myeloid cells 2” (TREM2) (4, 5, 6), of which there are several missense mutations in the ectodomain, including R47H, associated with increased risk for AD (4, 5, 6). The biological mechanisms underlying this association remain unclear.

Full-length TREM2 is expressed on the plasma membrane of microglia, where it can be cleaved by one or more metalloproteases to produce (i) a membrane-bound C-terminal fragment (CTF); and (ii) an N-terminal fragment consisting of the soluble ectodomain of TREM2 (sTREM2), which is released into the extracellular space (7, 8, 9). sTREM2 has been thought of as a nonfunctional, degradation product of TREM2 and used as a biomarker of microglial activation (10, 11, 12). However, several recent observations suggest the possibility that sTREM2 per se may play a role protecting against AD by interacting with Aβ. First, oligomeric Aβ binds to TREM2 and to sTREM2-Fc fusion protein (13, 14, 15), suggesting that sTREM2 might bind Aβ and potentially affect its aggregation state. Second, injection or expression of sTREM2 in the hippocampus of 5xFAD mice reduces both amyloid plaque load and memory deficits (16), indicating that sTREM2 inhibits amyloid pathology somehow. Third, in transgenic mouse models overproducing Aβ, the knockout of TREM2 expression accelerates amyloid plaque seeding (17) and the plaques have increased protofibrillar halos and hotspots (3, 16, 18), indicating that either TREM2 or sTREM2 inhibits plaque formation. Fourth, in the earliest presymptomatic stages of AD, at the time of Aβ deposition, sTREM2 levels in cerebrospinal fluid (CSF) are lower than in healthy controls (10, 19), consistent with sTREM2 being an endogenous inhibitor of Aβ deposition. However, the CSF Aβ levels rise in the early symptomatic stages of AD and then decline again at later stages of AD (11, 12). Fifth, mild cognitive impairment (MCI) and AD patients with higher sTREM2 levels in CSF have slower brain atrophy, cognitive decline, and clinical decline (20, 21), consistent with sTREM2 inhibiting AD progression. Sixth, healthy controls and MCI patients with higher sTREM2 levels in CSF have slower progression of amyloid and tau deposition (22), consistent with sTREM2 inhibiting Aβ aggregation and subsequent tau pathology.

All of these *in vivo* findings are compatible with the hypothesis that sTREM2 might protect against AD, potentially by impacting Aβ aggregation. To explore this hypothesis and the mechanisms involved, we investigated the interaction of Aβ with sTREM2 *in vitro*. We report that soluble Aβ oligomers bind TREM2 receptor on microglia and induce shedding of sTREM2. Next, we show that sTREM2 can bind and disaggregate Aβ oligomers, block Aβ fibrillization, and reduce Aβ neurotoxicity. These activities are attenuated in the R47H TREM2 holoprotein and R47H sTREM2. Moreover, the R47H sTREM2 promotes formation of morphologically distinct Aβ protofibrils. These data indicate additional mechanisms by which TREM2 protects against Alzheimer's disease and a previously unrecognized mechanism by which the R47H mutant increases risk.

## Results

### Aβ oligomers bind TREM2 holoprotein and induce TREM2 ectodomain shedding

To confirm that TREM2 can act as a cell surface receptor for Aβ oligomers, we treated mouse primary microglia or TREM2-transfected HeLa cells with Aβ42 oligomers that have been characterized both by electron microscopy and by oligomer/fibril specific antibodies (Fig. S1; note only Aβ42 was used in this work and will be referred to as Aβ henceforth). We then immunoprecipitated TREM2 from lysates of these cells and found that Aβ co-immunoprecipitated with endogenous TREM2 on primary microglia from wild-type mice, but not on microglia from TREM2^−/−^ knockout mice (Fig. S2*i*). The Aβ binding was prevented by TREM2-blocking antibody (Fig. S2*ii*). Oligomeric Aβ bound to TREM2 but not TREM1 (Fig. S2*iii*). Monomeric Aβ co-immunoprecipitated with TREM2 less efficiently than oligomeric Aβ (Fig. S2, *iv* and *v*, *p* = 0.03). The TREM2: Aβ oligomer interaction is at least partially specific because neither of two other neurodegeneration-associated oligomeric proteins (oligomeric α-synuclein or oligomeric Tau) bound to TREM2 even in HeLa cells overexpressing TREM2 and DAP12 (Fig. S3). These results confirm and extend the work of other groups showing that oligomeric Aβ binds TREM2 (13, 14, 15).

**Figure 1:**
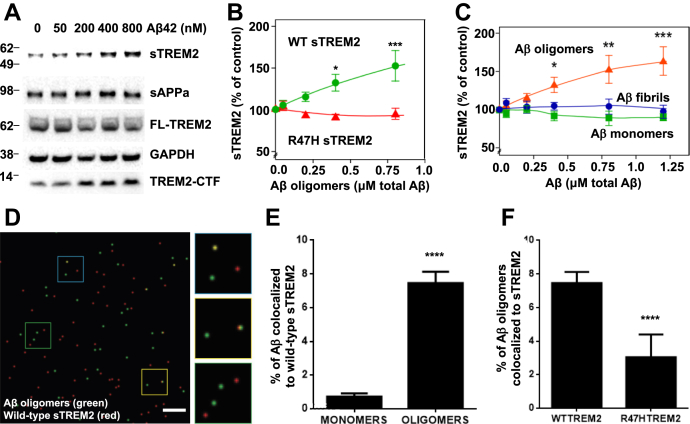
**Aβ oligomers induce TREM2 proteolysis and sTREM2 release, which then binds Aβ oligomers, but R47H sTREM2 binds less**. *A*, western blot of cell lysate and unprocessed supernatant (sTREM2) of HEK293 cells coexpressing human DAP12 and full-length N-terminally-tagged wild-type (WT) human TREM2 (FL-TREM2) 16 h after adding Aβ oligomers. This blot and those for Aβ monomers and fibrils are reproduced in Figure S5 for comparison. *B*, quantification of sTREM2 release from transfected HEK293 cells expressing wild-type (*green line*) and R47H TREM2 (*red line*). *C*, quantification of sTREM2 release from wild-type TREM2 expressing HEK293 cells induced by doses of Aβ oligomers (*red line*), monomers (*green line*), or fibrils (*blue line*). For both (*B* and *C*) error bars = SEM; ∗*p* < 0.05 ∗∗*p* < 0.01 ∗∗∗*p* < 0.001, n = 3 independent experiments; one-way ANOVA with Tukey's post-hoc multiple comparisons test. *D*, example field of single-molecule TIRF imaging of mixture of Aβ oligomers (*green*) and wild-type TREM2 ectodomain (*red*), where colocalized spots appear *yellow*. Scale bar: 1 micron. Magnified image of three sections of field at *right*. *E*, proportion of monomeric or oligomeric Aβ colocalized with wild-type sTREM2. *F*, proportion of Aβ oligomers colocalized with wild-type or R47H TREM2 ectodomain. For (*E* and *F*), error bars = SEM; ∗∗∗∗*p* < 0.0001, n = 3 independent preparations, each analyzed in nine fields each; two-tailed *t*-test of significance.

We next tested the consequences of Aβ oligomers binding to TREM2 on primary microglia. Aβ oligomers induced release of sTREM2 into the medium of primary microglia from wild-type mice, but not microglia from mice engineered to express R47H TREM2 (Fig. S4*i*, *p* < 0.05). To study this effect in more detail, we stably expressed human TREM2 (together with human DAP12) in HEK293 cells. Treatment of these cells with Aβ oligomers resulted in dose-dependent release of sTREM2 into the medium and the accumulation of TREM2-CTF in cell lysates (Fig. 1, *A* and *B* & Fig. S4*ii*). In contrast, treatment with Aβ monomers or fibrils resulted in no modulation of sTREM2's release (Figs. 1*C* and S5). We have previously shown that this shedding event occurs rapidly, within 1 h of Aβ oligomer binding, but that FL-TREM2 levels remain constant due to ongoing new synthesis of FL-TREM2 (8). The release of sTREM2 induced by oligomeric Aβ was attenuated in HEK293 cells expressing R47H TREM2 (Fig. 1*B* & Figs. S4*ii* and S5). Thus, oligomeric Aβ stimulates release of sTREM2 (potentially by endoproteolysis) from cells expressing wild-type TREM2, but less so from cells expressing R47H TREM2.

### Wild-type sTREM2 binds Aβ oligomers better than R47H TREM2

Because Aβ oligomers bind to TREM2 and induce shedding of sTREM2, it is of interest whether sTREM2 itself binds Aβ oligomers. Three groups reported that wild-type sTREM2 fused to the Fc domain of IgG binds oligomeric Aβ (13, 14, 15). However, Zhao *et al.* (13) and Zhong *et al.* (14) found that R47H sTREM2-Fc bound less than wild-type to Aβ, while Lessard *et al.* (15) found that they bound equally. We applied three orthogonal assays using an approach that did not require fusion of sTREM2 to the Fc domain of IgG, to reassess this question. Firstly, we examined this interaction at the molecular level using single-molecule total internal reflection fluorescence (TIRF) microscopy-based two-color coincidence detection (23). HiLyte 488-dye labeled Aβ was oligomerized and TAMRA-dye labeled sTREM2 added and imaged. Monomeric Aβ bleaches rapidly, so colocalization of Aβ and sTREM2 before and after bleaching enabled us to distinguish between monomers and oligomers. These experiments revealed that wild-type sTREM2 bound oligomeric Aβ much more than monomeric Aβ (Fig. 1, *D* and *E*) and that R47H sTREM2 bound less well to oligomeric Aβ than wild-type sTREM2 did (Fig. 1*F*). Second, this result was confirmed by a semiquantitative dot blot assay, which showed that wild-type sTREM2 preferentially bound oligomeric Aβ over monomeric Aβ and that R47H sTREM2 bound less of both forms of Aβ (Fig. S6, *i* and *ii*).

Third, we used Bio-Layer Interferometry (BLI) (24) to quantitatively assess the Aβ oligomer: sTREM2 interaction by immobilizing biotin-Aβ oligomers on Streptavidin biosensors and then exposing them to sTREM2. These studies suggested that sTREM2 associated with Aβ oligomers with two different rates and dissociated from Aβ oligomers with two different rates (Fig. S6, *iii* and *iv*), consistent with sTREM2 binding to different Aβ oligomer sizes with different affinities. Best fit association and dissociation curves for both wild-type and R47H sTREM2 were with a 2:1 heterogeneous ligand model. In agreement with the semiquantitative experiments described above, these BLI studies confirmed that R47H sTREM2 bound Aβ oligomers less well than wild-type sTREM2 (Table S1: TREM2 WT K_D_1 = 2.00 ± 0.15 μM, K_D_2 = 0.29 ± 0.08 μM; TREM2 R47H mutant K_D_1 = 11.70 ± 5.97 μM, K_D_2 = 1.22 ± 0.30 μM; where K_D_1 is the dissociation constant of the predominant interaction within the heterogeneous population of Aβ species).

### Wild-type sTREM2 inhibits Aβ oligomerization and dissolves Aβ oligomers, whereas R47H sTREM2 induces Aβ protofibrils

**Figure 2:**
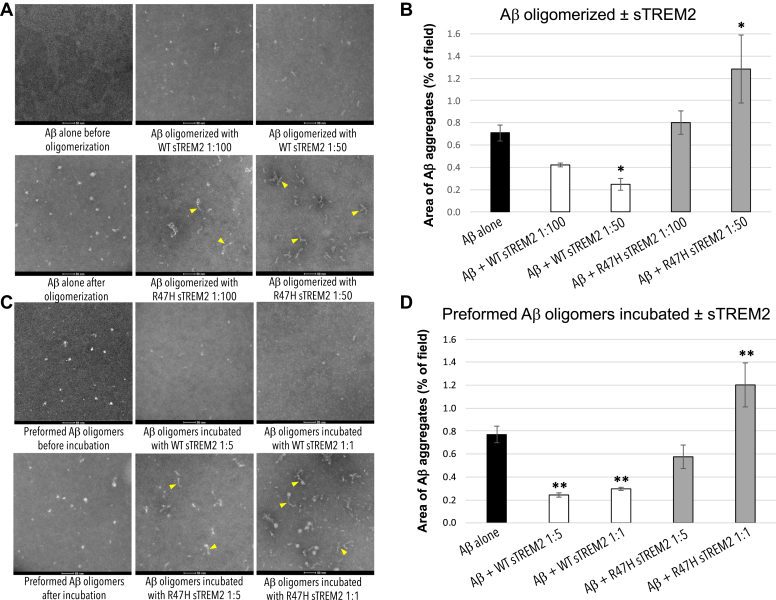
**Wild-type sTREM2 inhibits Aβ oligomerization and disaggregates A**β **oligomers, whereas R47H sTREM2 converts Aβ to protofibrils.***A*, Aβ was aggregated for 3 h at 37 °C ± WT sTREM2 or R47H at molar ratios of 1:100 or 1:50 (sTREM2: Aβ). TEM images revealed that WT sTREM2 reduced Aβ oligomerization, whereas R47H sTREM2 increased protofibrils (indicated by *yellow arrowheads*) and amorphous oligomers. *B*, quantification of three experiments indicated that WT sTREM2 decreased and R47H increased area of Aβ aggregates. *C*, preformed Aβ oligomers were treated with WT sTREM2 or R47H at molar ratios of 1:5 or 1:1 for 30 min. TEM images revealed that preformed Aβ oligomers were disaggregated by WT sTREM2 but formed more globular Aβ oligomers and protofibrils with R47H sTREM2. *D*, quantification confirmed decreased area of Aβ aggregates with WT sTREM2 and increased area with R47H sTREM2. For both (*B* and *D*), error bars represent SD, and statistical analysis was performed using one-way ANOVA followed by Bonferroni's multiple comparison test (n = 3 for each, ∗*p* < 0.05 *versus* Aβ oligomer, ∗∗*p* < 0.01 *versus* preformed Aβ oligomers).

Because sTREM2 bound to Aβ oligomers, we next investigated whether sTREM2 affected Aβ oligomerization. We incubated 22 μM monomeric Aβ ± 0.22 or 0.44 μM sTREM2 (wild-type or R47H) for 3 h at 37 °C, *i.e.*, conditions known to generate Aβ oligomers. The formation of Aβ oligomers was then assessed by transmission electron microscopy (TEM) (Figs. 2 and S7); dot blotting with A11 antioligomer antibody; and by high-performance liquid chromatography size-exclusion chromatography (HPLC-SEC) experiments (Fig. S8). These experiments revealed that preincubation of Aβ monomers with wild-type sTREM2 (at molar ratios of 1:50 and 1:100 sTREM2: Aβ) inhibited Aβ oligomerization (Fig. 2*A*) quantified by area (Fig. 2*B*) or number (Fig. S7*i*). SEC and antibody dot blots confirmed that sTREM2 inhibited formation of Aβ oligomers (Fig. S8*i*). Analysis of the size distribution of Aβ oligomers by TEM revealed that wild-type sTREM2 reduced the abundance of small oligomers (oligomer size <60 nm^2^) and eliminated larger oligomers (oligomer size >70 nm^2^) (Fig. S9*i*).

In contrast, R47H sTREM2 did not block Aβ oligomerization, but instead promoted the formation of morphologically-distinct Aβ protofibrils (Fig. 2, *C* and *D*; Figs. S7*ii* and S9*ii*). Thus, the presence of wild-type sTREM2 reduces the formation of Aβ oligomers, particularly larger oligomers, and in stark contrast, R47H sTREM2 induces Aβ monomers to form Aβ protofibrils.

To study reversal of oligomerization, preassembled Aβ oligomers were diluted to 2 μM and mixed with wild-type or R47H sTREM2 (at molar ratios of sTREM2: total Aβ of: 1:5 and 1:1) and incubated for 30 min at 37 °C. The Aβ assemblies were then assessed using the same methods as above. This experiment revealed that wild-type sTREM2 disaggregated preformed Aβ oligomers (TEM in Fig. 2, *C* and *D* and Fig. S7*ii*; anti-A11 dot blot and HPLC-SEC assays in Fig. S8*ii*). Wild-type sTREM2 strongly reduced the abundance of all but the very smallest oligomers (Fig. 2*C* & Fig. S9*ii*). In contrast, R47H sTREM2 increased the abundance of Aβ oligomers and induced the formation of morphologically-distinct Aβ protofibrils (Fig. 2*C* & Figs. S7*ii* and S9*ii*). Aβ protofibrils are large, linear Aβ oligomers, formed from Aβ monomers in a variety of conditions (25, 26), and are observed in CSF of MCI and AD patients but not healthy controls (27).

We repeated these experiments at lower concentrations, using preformed Aβ oligomers at 100 nM Aβ (monomer equivalent) incubated with 20 or 100 nM sTREM2. Wild-type sTREM2 (at either concentration) induced rapid dissolution of the Aβ oligomers (Figs. S10 and S11). In contrast, treatment with R47H sTREM2 induced aggregation of Aβ oligomers into much larger Aβ assemblies (Figs. S10 and S11).

### Wild-type sTREM2 inhibited and reversed Aβ fibrillization, whereas R47H was less effective

**Figure 3:**
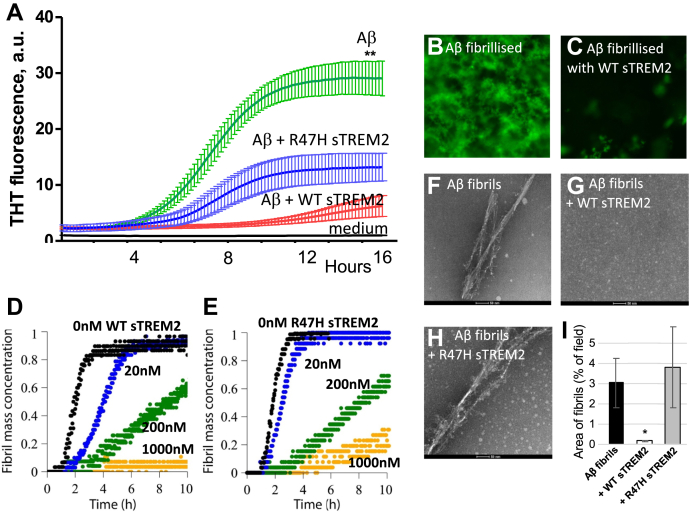
**Wild-type sTREM2 blocks Aβ fibrillization, but R47H inhibits less**. *A*, 10 μM of monomeric Aβ was incubated at 30^o^C in DMEM/F12 with 10 μM thioflavin T (THT) ± 1 μM wild-type or R47H sTREM2, and the fluorescence followed over time. Means and SD of three separate experiments are shown. There was significant difference (*p* < 0.01, ∗∗) between the final fluorescence of the Aβ v Aβ + WT sTREM2 samples. At the end of the assay, (*B*) in the absence of sTREM2, and (*C*) presence of wild-type sTREM2, the bottom of the well was imaged using a fluorescence microscope with a ×40 objective. *D*, 2 μM of monomeric Aβ was incubated at 37 °C in phosphate buffer with 10 μM THT +0, 0.02, 0.2, or 1.0 μM wild-type sTREM2, and the fluorescence followed over time. *E*, 2 μM of monomeric Aβ was incubated at 37 °C in phosphate buffer with 10 μM THT +0, 0.02, 0.2, or 1.0 μM R47H sTREM2, and the fluorescence followed over time. Preformed Aβ fibrils were either (*F*) untreated, (*G*) treated with WT sTREM2, or (*H*) treated with R47H sTREM2 at molar ratios of 1:1 and TEM imaged after 2 h. *I*, quantification of the area of Aβ fibrils confirmed that WT sTREM2 decreased Aβ fibrils, and R47H sTREM2 had no effect. Images (*F–H*) and data (*I*) are reproduced in Figure S12 with those for 30 min and 24 h for comparison. Error bars represent SD. Statistical analysis was performed using one-way ANOVA followed by Bonferroni's multiple comparison test (n = 3–8, ∗*p* < 0.05 *versus* preformed Aβ fibril).

Because wild-type sTREM2 inhibited Aβ oligomerization and disaggregated Aβ oligomers, we decided to test whether sTREM2 affected Aβ fibrillization. In the absence of sTREM2, Aβ (10 μM) fibrillized with standard lag-phase kinetics as measured with Thioflavin T fluorescence. However, 1 μM of wild-type sTREM2 almost completely prevented Aβ fibrillization (Fig. 3, *A*–*C*). In total, 1 μM of R47H sTREM2 did not prevent Aβ fibrillization, but the Thioflavin T fluorescence was lower (Fig. 3*A*), suggesting fibrils with less β sheet structure. Repeating these experiments, at lower concentrations and higher temperature, gave similar results. Thus, fibrillization of 2 μM Aβ was substantially delayed by 20 nM sTREM2 (*i.e.*, a 100:1 ratio) and prevented by 1 μM sTREM2 (Fig. 3*D*). In contrast, at the same low dose (20 nM), R47H sTREM2 had minimal effect on Aβ fibrillization (Fig. 3*E*).

To test whether sTREM2 could disaggregate fibrils of Aβ, small fibrils were preassembled and diluted to 2 μM (monomer equivalent) and mixed with wild-type or R47H sTREM2 (at molar ratios of sTREM2: total Aβ of 1:1) and incubated for 30 min, 2 h, or 24 h at 37 °C. Wild-type sTREM2 induced rapid and complete disaggregation of the preformed Aβ fibrils, whereas R47H sTREM2 had little effect on fibrils (Fig. 3, *G–I*; Fig. S12), although there may be an increase in oligomers (Fig. 3*H*).

### Wild-type sTREM2 inhibits Aβ-induced membrane permeabilization and neurotoxicity, while R47H sTREM2 increases Aβ-induced neurotoxicity

Because sTREM2 bound Aβ oligomers and inhibited Aβ oligomerisation and fibrillization, we next tested whether sTREM2 affected the neurotoxicity of Aβ. Aβ neurotoxicity is thought to be mediated by two different mechanisms—namely by permeabilization of neuronal membranes (28) and by microglial activation (28, 29).

**Figure 4:**
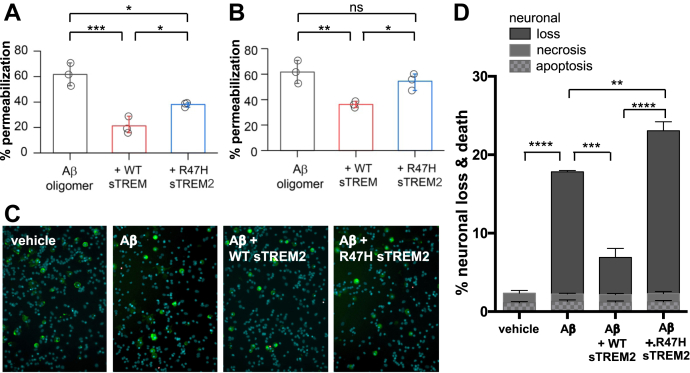
**Wild-type sTREM2 blocks Aβ-induced membrane permeabilization and neuronal loss, but R47H sTREM2 inhibits less. *A*,** 10 μM of monomeric Aβ was aggregated ±1 μM wild-type (WT) or R47H human sTREM2 for 6 h, diluted to 200 nM, and membrane permeabilization assay was performed. Error bars = SD of three independent experiments; ∗∗*p* < 0.01; statistics: two-sample *t*-test. *B*, 10 μM of monomeric Aβ was aggregated for 6 h, diluted to 200 nM, then incubated for 30 min with vehicle, 200 nM WT sTREM2 or R47H sTREM2, before the membrane permeabilization assay was performed. Error bars = SD of three independent experiments; ∗∗*p* < 0.01; statistics: two-sample *t*-test. *C* and *D*, mixed neuronal–glial cocultures were treated with either: vehicle, 250 nM monomeric Aβ, 250 nM Aβ + 25 nM WT sTREM2 or 250 nM Aβ + 25 nM R47H sTREM2. Three days later, cultures were stained with isolectin B4 (*green*, to identify microglia), propidium iodide (*red*, to identify dead cells), and Hoechst 33342 (*blue*, to identify cells and whether apoptotic) and imaged (representative fields: *C*), and the number of apoptotic, necrotic, and healthy neurons were quantified (mean data: *D*). Loss is the decrease in neuronal density relative to vehicle-treated cultures. Error bars = SEM; ∗∗*p* < 0.01, ∗∗∗*p* < 0.001, ∗∗∗∗*p* < 0.0001; n = 4 independent experiments on separate cell cultures. Statistical analysis was by one-way ANOVA and Tukey's post-hoc test.

Consequently, we initially used nanosized phospholipid membrane vesicles containing a calcium-sensitive fluorophore to explore the ability of Aβ to permeabilize membranes. Prior work has shown that Aβ oligomers (but not monomers or fibrils) can permeabilize these membranes (28). We found that wild-type sTREM2 and R47H sTREM2 proteins themselves had no effect on membrane permeabilization when added in the absence of Aβ (Fig. S13*i*). By contrast, Aβ oligomerized for 6 h in the absence of sTREM2 induced permeabilization of the vesicles. However, Aβ oligomerized in the presence of wild-type sTREM2 (at molar ratios of sTREM2: total Aβ of 1:10) induced significantly less permeabilization (Fig. 4*A*). Wild-type sTREM2 also inhibited the permeabilization induced by preformed Aβ oligomers (at a 1:1 M ratio) (Fig. 4*B*). The simplest explanation for this inhibition of permeabilization is our previous finding that wild-type sTREM2 inhibits Aβ oligomerization and disaggregates Aβ oligomers at these concentrations. However, the binding of wild-type sTREM2 to Aβ oligomers might also reduce their toxicity.

When these experiments were repeated using R47H sTREM2 (at a molar ratio of sTREM2: total Aβ of 1:10), we found that Aβ oligomers formed in the presence of R47H sTREM2 also induced less permeabilization than Aβ oligomerized alone but allowed more permeabilization than Aβ oligomerized with wild-type sTREM2 (Fig. 4*A*). In contrast to wild-type sTREM2, R47H sTREM2 did not significantly inhibit permeabilization induced by preformed Aβ oligomers (Fig. 4*B*). These results are consistent with wild-type sTREM2 reducing Aβ oligomer abundance but R47H sTREM2 not reducing Aβ oligomer abundance (Fig. 2).

We next tested whether sTREM2 could block the neurotoxicity induced by Aβ in neuronal–glial cocultures. We have previously shown that 250 nM Aβ induces slow, progressive neuronal loss mediated by microglia in this coculture system (29). We found that 250 nM Aβ induced neuronal loss over 3 days, and this Aβ-induced neuronal loss was substantially reduced by cotreatment with 25 nM wild-type sTREM2 (Fig. 4, *C* and *D*). In contrast, cotreatment of the cultures with 25 nM R47H sTREM2 increased the neuronal loss above the level induced by 250 nM Aβ alone (Fig. 4*D*). Microglial density was not affected by the treatments; and wild-type and R47H sTREM2 proteins by themselves had no significant effect on neuronal loss (Fig. S13*ii*). As membrane permeabilization is thought to be mediated by smaller Aβ oligomers, while microglial activation is thought to be mediated by larger Aβ aggregates (28), our finding that R47H sTREM2 increased Aβ-induced neuronal loss (Fig. 3*D*), which is known to be microglia-mediated (29), is consistent with R47H sTREM2 increasing larger Aβ aggregates (Fig. 2). However, we can't rule out sTREM2 acting directly on the microglia (16) to modify neurotoxicity. Wild-type sTREM2 may also be protecting by simply binding Aβ oligomers, preventing their neurotoxicity, but at the molar ratios, concentrations, and measured affinities of sTREM2 and Aβ oligomers, the proportion of Aβ oligomers bound to sTREM2 is likely to be small in the conditions used, so the contribution of this mechanism to neuroprotection is likely to be small. However, in order to distinguish between these mechanisms more clearly, we tested whether WT sTREM2 could prevent neuronal loss induced by oligomeric Aβ at a molar ratio of 1:100 (sTREM2:Aβ monomer equivalents) conditions under which sTREM2 could not feasibly bind all the Aβ oligomers and found that WT sTREM2 still prevented the neuronal loss (Fig. S14), consistent with sTREM2 protecting by disaggregating Aβ oligomers. Overall, our results indicate that wild-type sTREM2 reduces Aβ neurotoxicity, while R47H TREM2 reduces the direct Aβ neurotoxicity less than WT sTREM2 and increases the Aβ neurotoxicity in cultures where microglia are present.

## Discussion

We have shown that Aβ oligomers, but not Aβ monomers or fibrils, bind to microglial TREM2 and induce sTREM2 release, and this released sTREM2 also preferentially binds to Aβ oligomers, consistent with previous reports (13, 14, 15), (although Lessard *et al.* (15) did not find sTREM2 release in response to Aβ oligomers). Crucially, however, we also found that wild-type sTREM2 inhibits the formation of fibrils and larger Aβ oligomers and disaggregates protofibrils and larger Aβ oligomers into the smallest Aβ oligomers. This is consistent with a recent, preliminary report (30) that WT sTREM2 inhibited Aβ aggregation, measured by thioflavin T assay. In contrast, we found that R47H sTREM2 promoted the formation of Aβ protofibrils, indicating a gain of function by this mutation. These effects may provide a partial explanation of how a single copy of the TREM2 R47H mutant is associated with increased risk for AD, by increasing production of neurotoxic forms of Aβ.

One potential caveat is the protein concentrations used in our experiments. In order to observe Aβ aggregation over a reasonable experimental timeframe, the lowest concentration of Aβ used here was 100 nM, whereas levels found in CSF are about 0.1 nM (10, 20, 31). However, the Aβ oligomer concentration near amyloid plaques was estimated as 700 nM (32), and plagues are likely be a more relevant location for Aβ aggregation in AD than CSF. sTREM2 concentrations in lumbar CSF average about 0.2 nM (10, 20, 31); however again, it is likely that sTREM2 levels are higher near amyloid plaques surrounded by activated microglia, which express high levels of TREM2 (3). Note also that the observed effects of sTREM2 on Aβ are rapid: 20 nM sTREM2 dissolved 100 nM Aβ oligomers within 30 min *in vitro* (Fig. S11). Lower concentrations are likely to have the same disaggregating effect over a slower time course, which may be more relevant to the slow time course of AD.

We did not investigate the molecular mechanisms by which sTREM2 affected Aβ aggregation, but our finding that wildtype sTREM2 stabilizes the smallest observable oligomers (Figs. S9 and S11) suggests the possibility that sTREM2 binds preferentially to these (or undetectable oligomers such as dimers), thereby potentially blocking further growth of aggregates and disaggregating larger Aβ aggregates into smaller, potentially nontoxic forms. Precedent for this is the finding that α-synuclein-specific single-domain antibodies (nanobodies) bind α-synuclein stable oligomers and convert them into less stable oligomers with reduced toxicity (33). However, clearly more research is required to determine the mechanisms for sTREM2 and Aβ.

In summary, our experimental data reveal how Aβ oligomer binding to TREM2 may mediate direct (*via* activation of intracellular TREM2-dependent signaling pathways) and indirect protective mechanisms (*via* effects on Aβ oligomer assembly and toxicity). Our studies are broadly congruent with previous research showing that knockout of TREM2 expression in APP mice resulted in accelerated amyloid plaque seeding (17), with increased protofibrillar halos and hotspots around these plaques (3, 16, 18). Discrepant results were published for R47H TREM2 knockin mice (34), but these mice have been found to have little or no R47H TREM2 expression (35). However, these authors did show that WT sTREM2 bound strongly to amyloid plaques (34), consistent with our results. Our studies provide a potential explanation of the clinical observation of slower rates of cognitive and clinical decline in patients with MCI or AD who have higher levels of sTREM2 in the CSF (20, 21) and slower rates of amyloid deposition in healthy controls and MCI patients with higher sTREM2 levels in CSF (22). They also provide a potential explanation for the beneficial effects of infusing or expressing sTREM2 into the brain of mouse models of AD (16). Additional biophysical studies will be required to identify the key Aβ species dynamically interacting with wild-type sTREM2 to prevent neurotoxicity, and the Aβ species stabilized by sTREM2 R47H to increase neurotoxicity. This knowledge could be exploited for the design of small brain-penetrant molecular mimics of sTREM2 as potential therapeutics for AD.

## Experimental procedures

### cDNA constructs

Wild-type human TREM2 (hTREM2) and DAP12 (hDAP12) were subcloned in pCMV6-A vector (Origene). A single C→A nucleotide polymorphism (SNP) at codon 85 (T85K) on the Origene TREM2 cDNA clone was reverted to consensus wild-type sequence by site-directed mutagenesis.

### Antibodies

The following antibodies were used: mouse TREM2 (#AF1729, targeting on N-terminal TREM2, 1:500 for western blot; R&D); DDK (FLAG) (#TA50011, 1:2000 for western blot; Origene); V5 (#46-0705, 1:2000 for western blot; Life Technologies, Burlington); Nicastrin (#sc-14369, 1:200 for western blot; Santa Cruz); GAPDH (#2118, 1:3000 for western blot; Cell Signaling); Anti-His (C-term)-HRP (#46-0707, 1:2000 for dot blot, Invitrogen); Aβ (6E10, 1:1000 for western blot and dot blot, Covance); TrueBlot secondary antibodies conjugated with horseradish peroxidase (anti-goat IgG, anti-mouse IgG, and anti-sheep IgG, 1:1000 for western blot after immunoprecipitation; Rockland); secondary antibodies conjugated with horseradish peroxidase (anti-mouse IgG; anti-rabbit IgG, 1:2000 for western blot; Thermo Scientific). Function-blocking TREM2 antibody (Clone 78.18, 25 μg/ml for blocking assay, AbD Serotec catalogue number MCA4772EL) and a control antibody (Rat IgG1, AbD Serotec) were used for blocking experiments.

### Animals

All experiments were performed in accordance with Canadian Council on Animal Care guidelines and UK Animals (Scientific Procedures) Act (1986) and approved by the Animal Care Committee at the University of Toronto and the Cambridge University local ethics committee.

TREM2-deficient mice were obtained from Dr J. Gommerman and were constructed by targeted homologous recombination (36), which removed Exon 1 and 2, which include the start codon and the major extracellular IgG domain. In contrast to the recently reported VelociGene construct, the direction of the Hygromycin cassette was “reversed.” Crucially, in agreement with two other models, but in contrast to the VelociGene construct, RT-PCR analyses confirmed specific loss of TREM2 expression without the perturbation of TREM1L expression observed in the VelociGene construct (36).

CRISPR-Cas 9 genome editing was used to create mouse models with mutations in Trem2 (R47H) at the Toronto Centre for Phenogenomics (37). Guide RNAs for each of the CRISPR genomic edits were assessed by two different programs for off-target sites (38, 39). Repair templates were designed to result in the desired amino acid substitution and to optimize or maintain comparable codon usage. The Trem2 R47H lines were validated by whole gene sequencing, RT-PCR, and western blotting. After breeding with C57BL/6 mice, heterozygous R47H/+ mice were then used to generate homozygous lines from each of the two founders. All genotyping was performed by genomic DNA sequencing. As with other similar CRISPR-Cas9 engineered mouse lines, this construct results in altered splicing and reduced expression (35), and consequently, we have not focused on the cell biology of the R47H microglia.

**Table:** 

Gene edit	Guide RNA and location (Chr and strand)	Repair template	Primers
Trem2 R47H	gRNA-3 ACTGGGGGAGACGCAAGGCC Chr17:48548344 (+1)	CTGCAGGGCATGGCCGGCCAGTCCTTGAGGGTGTCATGTACTTATGACGCCTTGAAGCACTGGGGGAGACaCAAGGCCTGGTGTCGGCAGCTGGGTGAGGAGGGCCCATGCCAGCGTGTGGTGAGCACACACGGTGTGTGGCTGC	Trem2 PCR_F: GTGGAAGGTACCCAAGGACC Trem2 PCR_R: CAGGCACCTACAAGTAGCCC Trem2 Seq_F: TCTCCTCTGCAGCCCTGTC Trem 2 Seq_R: AGCTACTGTGTACTCACCCTC.

### Primary microglial cell culture

Primary cultures of mixed glial cells and pure microglia were prepared from the cerebral cortex of P0-3-day-old C57BL/6 TREM2^+/+^ or TREM2^−/−^ mice as described (40). After removal of olfactory bulbs, cerebral hemispheres were cut into ∼1 mm pieces, vortexed for 1 min, filtered through a 40 μm cell strainer, and plated in 80 cm^2^ tissue culture flasks. Mixed glia were cultured until microglia appeared (10–18 days after plating). Microglia were isolated by shaking flasks. Purified microglia were replated for analysis. On average, each experiment used microglia from 30 to 35 pups. All pups used in this study (wild type; R47H, and TREM2KO) were maintained on a C57BL/6 background.

### Mixed neuronal–glial cocultures and treatments

Mixed neuronal–glial cocultures were prepared from cerebella of 5–7-day-old rats (40), cultured for 7 days, then treated with either: vehicle, 250 nM monomeric amyloid beta 1–42 (Aβ), 250 nM Aβ + 25 nM wild-type sTREM2, or 250 nM Aβ + 25 nM R47H sTREM2 for 3 days. Three days later, neuronal death and loss were quantified by staining the live cultures with propidium iodide for necrosis, with isolectin IB4 for microglia, and with Hoechst 33342 for nuclei, which is used to distinguish healthy (uncondensed) from apoptotic (condensed) nuclei and to distinguish astrocytes (large bean-shaped nuclei) from neurons and microglia (small, round nuclei). Neurons are easily distinguished from astrocytes (by morphology) and from microglia (IB4 only binds microglia in these cultures), and the apoptotic neurons undergo strong nuclear condensation identified using the Hoechst 33342 staining (29, 40). The number of apoptotic, necrotic, and healthy neurons, astrocytes, and microglia are then counted on photos of multiple set fields, as previously described (29, 40). These cultures are 85 ± 5% neurons, 7 ± 3% astrocytes, and 5 ± 3% microglia.

### Amyloid β-peptide

Human Aβ42 (used in TREM2 cleavage), HiLyte Fluor 488-labeled Aβ42 (used in single-molecule TIRF imaging), HiLyte Fluor 647-labeled Aβ42 (used in IP and dot blot), and Biotin-Aβ42 (used in BLI) were purchased from AnaSpec and Bachem; Aβ42 oligomers were prepared as endotoxin-free preparations (41) and validated by antibodies, gels, and electron microscopy (Fig. S1*i*). Prior work has established that these fluorescent tags do not significantly impact Aβ assembly (42).

### Recombinant sTREM2 expression and purification

cDNAs encoding residues 19–143 of WT and R47H TREM2 were ordered as linear DNA strings (Life Technologies) and inserted into pHLSec. The plasmids were transfected into Freestyle 293-F cells (Life Technologies) grown in suspension in FreeStyle293 medium. Cells were transfected at a density of 2.5 × 10^6^/ml with a DNA concentration of 3 μg/ml and polyethyleneimine (Linear PEI 25 kDa molecular weight, PolySciences Inc) at 9 μg/ml. On day 6 posttransfection, conditioned medium was harvested and TREM2 protein was purified using IMAC (Histrap excel, GE Healthcare) followed by gel filtration chromatography in storage buffer—20 mM HEPES, 200 mM NaCl, pH 7.0. The purity of WT and R47H sTREM2 was assessed by SDS-PAGE gels stained with Coomassie, which revealed only a single band for each (Fig. S15). To maintain a low endotoxin level: endotoxin-free chemicals and plasticware were used; all chromatography media, columns, and concentrators (Vivaspin Turbo) were soaked for >12 h in 1 M NaOH. Proteins in storage buffer were concentrated to between 5 and 10 mg/ml for use and tested for endotoxin with the EndoZyme recombinant Factor C assay (Hyglos GmbH). Endotoxin levels in all assays were maintained at < 0.1 EU/ml.

### Co-immunoprecipitation and western blot

Primary microglia were rinsed with ice-cold Dulbecco's mG,odified Eagle medium without phenol red and incubated with 100 nM HiLyte Fluor 647-labeled Aβ at 4 °C for 1 h. For antibody blocking assays, before Aβ incubation, microglia cultures were preincubated with 25 μg/ml monoclonal anti-TREM2 antibody or rat IgG1 control for 20 min Cells were collected by scraping, washed with ice cold 1xPBS, and centrifuged. Primary microglia were lysed with 1% CHAPSO buffer (1% 3-[(3-cholamydopropyl) dimethylammonio]-2-hydroxy-1-propanesulfonate (CHAPSO), 25 mM HEPES, 150 mM NaCl, 2 mM EDTA, pH 7.4). Crude membrane fractions were extracted from HeLa cell pellets as described (43) and lysed with 1% CHAPSO buffer. Primary antibodies and corresponding IgG controls (1 μg/reaction) were incubated with lysates at 4 °C for 2 h, then mixed with Protein G Dynabeads (Life Technologies), incubated at 23 °C for 20 min, then washed and eluted as per manufacturer's instructions. The immunoprecipitates were separated on 4–12% MES/Bis-Tris gels (Life Technologies). Fluorescent signals of Aβ were detected using an Odyssey FC imaging system (LI-COR). Proteins were transferred onto nitrocellulose membranes, which were probed with primary antibodies and corresponding TrueBlot secondary antibodies.

*In vitro* coprecipitation of TREM2 or TREM1 with Aβ oligomers was performed by mixing recombinant Fc-tagged TREM2 or TREM1 ectodomain (residues 19–171; 100 ng/ml, R&D systems), 100 nM HiLyte Fluor 647-labeled Aβ oligomers, and Protein G Dynabeads in 0.5% fatty acid free BSA PBS-Tween buffer. The mixture was incubated at room temperature for 1 h and then washed, eluted, and analyzed the same as described above.

### Dot blot

Aβ monomers or oligomers (1 μg/dot) prepared as described above and anti-TREM2 antibody (0.3 μg/dot) were spotted onto a nitrocellulose membrane. Membrane strips were blocked with 3% fatty acid free BSA (Sigma Aldrich) and then incubated with 100 nM recombinant His-tagged WT/R47H TREM2 ECD diluted in blocking buffer at 4 °C for 1 h. Bound TREM2 ECDs were probed with HRP-conjugated anti-His tag antibody and detected with Odyssey FC imaging system.

### Bio-layer interferometory

#### Preparation of Aβ oligomers for Bio-Layer Interferometry (BLI) studies

Synthetic human Aβ residues 1–42 and biotinylated Aβ42 were purchased from Bachem or AnaSpec and solubilized in hexafluoroisopropanol (HFIP) with DMSO (200 μl) to generate monomeric species. Solubilized Aβ was diluted in filtered assay buffer (20 mM HEPES, 500 mM NaCl pH 7.4])containing DMSO (2%) to a total volume of 9800 μl and a peptide concentration of 0.1 mg/ml (22 μM). Peptide solutions were aliquoted and incubated for 3 h at 37 °C with stock solutions stored at –80 °C. TEM was performed using undiluted biotinylated Aβ (100%) or a mixture of biotinylated Aβ (10%) and unlabeled Aβ (90%), which were diluted to final concentrations of 1.1 μM (Fig. S1, *iii* and *iv*). These TEM samples (10 μl) were applied to carbonate coated grids and negatively stained with 1% phosphotungstic acid (PTA). TEM micrographs indicating the globular morphology of the Aβ oligomers were obtained on a Hitachi H-7000 operated at 75 kV. The biotinylated oligomers were indistinguishable from nonbiotinylated Aβ oligomers.

#### Binding affinity of TREM2 ectodomain to Aβ was analyzed by Bio-Layer Interferometry (BLI) using the Octet RED384 system (ForteBio)

Assays were performed at 30 °C in the assay buffer (20 mM HEPES, 500 mM NaCl, 0.1% w/v BSA, 0.02% v/v Tween20, pH 7.4) with vibration at 1000 rpm. Streptavidin biosensors loaded with Biotin-Aβ (Anaspec) were incubated with TREM2 ectodomain WT (1, 2, 5, 10 μM) or R47H (2, 5, 10, 20 μM) to obtain the association curves and subsequently in the assay buffer to obtain the dissociation curves. Kinetics data were analyzed using a 2:1 heterogeneous ligand model.

### Single-molecule TIRF imaging

#### Aggregation of Aβ for TIRF studies

Stock solutions of HiLyte Fluor 488- labeled Human Aβ42 (Aβ, 0.1 mg; AnaSpec) were prepared by dissolving the lyophilized peptide (1% NH_4_OH, 50 μl) followed by dilution into pH 7.4 PBS to 200 μM, aliquoted, and stored at –80 °C. Working solutions were prepared by diluting the monomeric stock solutions into pH 7.4 PBS on ice to the concentration used for the aggregation (0.5 μM). The working solution was then placed into a shaking incubator (3 °C, 200 rpm) for 6 h to ensure a significant population of oligomeric species. All protein samples were stored and diluted into LoBind microcentrifuge tubes (Eppendorf).

#### Preparation of slides for TIRF microscopy

Slides were prepared in the same way as previously described (23). Slides containing unlabeled Aβ and TREM2 were tested for fluorescence artefacts.

#### TIRF microscope setup

Colocalization experiments were performed on a bespoke TIRF setup. Continuous-wave solid-state lasers operating at 488 nm and 641 nm were used for imaging. The beam power was controlled by attenuation through a neutral density filter, following which undesirable wavelengths were filtered out through excitation filters (LL01-488-25, FF01-640/14-25, Semrock). The beams were then circularly polarised by a quarter wave plate. Laser lines were combined by dichroic mirrors. The beams were then passed through a Köhler lens into the back aperture of an inverted microscope body (Olympus, IX73) and were reflected by a dichroic mirror (Di01-405/488/532/635, Semrock) through an oil immersion objective (Olympus, 60XOTIRF). Emitted radiation was collected through the same objective and passed through the dichroic before being filtered (FF01-480/40-25, LP02-647RU-25, Semrock). The fluorescence emission was then projected onto an EMCCD camera (Evolve Delta, Photometrics), each pixel was 275 nm in length. Data visualization was achieved through Micro-Manager and ImageJ software.

#### Single-molecule TIRF imaging

The colocalization experiment was designed to investigate binding of Aβ oligomers with TREM2. The Aβ 6 h aggregation contained a mixture of monomeric and oligomeric species, hence illumination was performed for significant time periods to photobleach the monomeric species. Automated TIRF colocalization experiments were performed with the use of a custom script (BeanShell, micromanager). Each data set consisted of a 3 × 3 grid of nine images at different areas of the coverslide, distances between images was 350 μm as previously described (20). Images were recorded at 33 ms exposure, beginning with 800 frames excitation with 641 nm followed by 800 frames excitation, 488 nm excitation in the same field of view.

#### Colocalization analysis

Colocalization data was analyzed with a bespoke ImageJ macro. The 488 nm illumination channel contained a mixture of monomeric and oligomeric Aβ species, it was determined that the majority of monomeric species were photobleached by 40 frames of 488 nm illumination. Therefore, only frames after this point were considered in this analysis. The 488 nm and 641 nm illumination channels were compressed in time to create two single-frame images representing the average pixel intensities. Following which, points of intensity representing HiLyte Fluor 488 Aβ or AF647-TREM2 above a background threshold were located, counted, and binary images of these maxima were created. The two images were then summed to identify colocalized points. Chance coincident spots were excluded by 90° rotation of the binary image representing AF647-TREM2 points and summation with the HiLyte Fluor 488 Aβ image. Chance coincident spots were subtracted from the actual coincidence value, and percentage coincidence was calculated with the equation below:

$$\text{Coincidence}=\left(\frac{N_{A\beta -TREM2}}{\left(N_{A\beta -TREM2}-N_{A\beta }\right)}\right)\times 100$$

### TREM2 cleavage assays

#### HEK293 cell lines

HEK293 cells stably coexpressing HaloTag-WT hTREM2 and hDAP12 were grown on six-well plates for 48 h. Media were replaced by treatment media (1 ml of OptiMEM supplemented with 0.5% FBS) containing defined concentrations of freshly made Aβ42 oligomers or crude neuronal membrane lipid extracts. Cells were collected 16 h after treatment, and amounts of CTF-TREM2 were assayed by western blotting. NTF release in conditioned media was detected by specific binding of the fluorescently labeled HaloTag ligand, HaloTag TMR direct. In total, 20 μl of media was loaded on gel, and the NTF-related fluorescence was detected using the LI-COR Odyssey imager. Where indicated, HEK293 cells were transfected with human TREM2 tagged at the N-terminus or with R47H or WT human TREM2 (together with human DAP12), as previously described (8).

#### Primary microglia

Primary microglia from wild and TREM2 mutant mice were isolated from mixed glia culture by shaking and grown on 96-well plates for 24 h. Media were replaced by treatment media (1 ml of OptiMEM supplemented with 0.5% FBS) containing defined concentrations of freshly made Aβ oligomers. Cells were collected 24 h after treatment, and NTF release in conditioned media and TREM2 in cell lysate were detected by ELISA. TREM2 ELISA was performed on 96-well plates previously coated by TREM2 antibody (clone 78.18) and blocked with 0.5% BSA in 0.05% Tween20 in 1× PBS (PBST). Medium or cell lysate samples were added to precoated wells and incubated overnight at 4 °C. After washing with PBST, wells were incubated with TREM2 antibody (sheep anti-mouse TREM2, AF1729) diluted in blocking buffer for 2 h at room temperature. After secondary antibody (anti-sheep-HRP, 1:5000) incubation for 1 h at room temperature and washing, TMB chromogenic substrate was added to each well and developed at room temperature in dark for 20 min. The absorbance at 450 nm was read immediately after stop solution was added. TREM2 cleavage was represented by the ratio of relative values of sTREM2 in medium and total TREM2 in cell lysate, after subtracting the corresponding background values from TREM2 KO microglia in the same ELISA experiment.

### sTREM2 effects on Aβ oligomers

#### Preparation of Aβ oligomers and Aβ fibrils and sTREM2 treatment

HFIP-treated human synthetic Aβ42 was purchased from Bachem. Aβ solubilized by DMSO was diluted in PBS (pH 7.4) to a peptide concentration of 0.1 mg/ml (22 μM). To study sTREM2 effects on assembly of Aβ oligomers, monomeric Aβ (22 μM) solutions were incubated with or without WT sTREM2 or sTREM2 R47H (sTREM2: Aβ, 1:100 or 1:50) for 3 h at 37 °C. To study reversal of oligomerization, monomeric Aβ (22 μM) solutions were incubated for 3 h at 37 °C to make oligomers, then diluted and mixed with WT sTREM or sTREM2 R47H (sTREM2: Aß, 1:5 or 1:1) to a Aβ concentration of 2.2 μM. Then Aβ and sTREM2 mixture was incubated for 30 min at 37 °C. To study dissociation of fibrils, monomeric Aβ (110 μM) solutions were incubated for 24 h at 37 °C with shaking (150 rpm), then diluted and mixed with WT or R47H sTREM2 (sTREM2: Aβ, 1:1) to a Aβ concentration of 2.2 μM, then incubated for 30 min, 2 h, or 24 h at 37 °C. These peptide solutions were then used for TEM, dot blot, and HPLC-SEC experiments.

#### Transmission electron microscopy (TEM)

Aβ oligomers were prepared as described above. TEM was performed to characterize Aβ or mixtures of sTREM2 and Aβ, which were diluted to final Aß concentrations of 1.1 μM or 100 nM. These TEM samples (10 μl) were applied to carbonate-coated grids for 1 min and negatively stained with 1% phosphotungstic acid (PTA) for 1 min. TEM micrographs were obtained on a Hitachi H-7000 operated at 120 kV.

#### Dot blots

Aβ oligomers were prepared as described above. Aβ and mixtures of sTREM2 and Aβ oligomers (1 μg/dot) prepared as described above were spotted onto a nitrocellulose membrane. Membrane strips were blocked with 5% fatty acid free BSA (Sigma Aldrich) and then incubated with antioligomer-specific antibody (A11, ThermoFisher Scientific) or antiamyloid β sequence-specific antibody (4G8, Millipore) for 1 h at RT and probed with HRP-conjugated anti-rabbit or mouse IgG antibodies and detected with ChemiDoc XRS+ imaging system (Bio-Rad).

#### HPLC-SEC

Aβ oligomers were prepared as described above. The size of prepared Aβ peptides was analyzed by SEC. Samples were run on a BioSep SEC-S 4000 (Phenomenex) column using a ProStar 210 HPLC (Varian) system with a ProStar 325 UV detector (Varian). Injected samples were eluted with PBS (pH 7.4) at a flow rate of 0.5 ml/min at ambient temperature, and data were obtained at 280 nm. Peak areas were integrated using Star 6.2 Chromatography Workstation (Varian).

### Aβ fibrillization assay

In total, 10 μM of monomeric Aβ was incubated at 30^o^C in DMEM/F12 with 10 μM Thioflavin T ± 1 μM wild-type or R47H sTREM2 in Corning 96 Well White Polystyrene Microplate with a clear flat bottom in a FluoroSTAR Optima plate reader (with orbital shaking between readings) to monitor fluorescence. Alternatively, 2 μM of monomeric Aβ was incubated at 37 °C in 20 mM phosphate buffer (pH 8) with 10 μM Thioflavin T ± 0, 0.02, 0.2, or 1.0 μM wild-type or R47H sTREM2 in a fluorescence plate reader with a 440-nm excitation filter and a 480-nm emission filter without stirring as previously described (44).

### Membrane permeabilization assay

Lipid vesicles were prepared as previously described (45), using a 100:1 mixture of phospholipids 16:0–18:1 phosphatidylcholine and biotinylated lipids 18:1–12:0 Biotin phosphatidylcholine. The mean diameter of vesicles was ∼200 nm, and these were filled with 100 μM Cal-520 dye using five freeze-and-thaw cycles. Nonincorporated Cal-520 dye was separated from the dye-filled vesicles by SEC. The vesicles were immobilized in biotin-labeled PLL-g-PEG-coated glass coverslips using a biotin–neutravidin linkage. Then coverslips were incubated with 50 μl Ca^2+^ containing buffer solution and different position of the coverslips imaged (F_blank_). Stage movements were controlled using an automated bean BeanShell-based program, which allows fields-of-view selection without user bias. Then the 10 μM aggregation (6-h time point) solution was added to the coverslips so that the final concentration of the peptide under the coverslip was 200 nM. Then the sample was incubated with the vesicles for 20 min, and the same fields of view of each coverslip were imaged (F_sample_). Then, 10 μl of 1 mg/ml of ionomycin was added to the same coverslips, and the same fields of view were imaged again (F_ionomycin_). Ionomycin is a calcium ionophore and causes saturation of the fluorescence intensity observed. This allows us to normalize fluorescence originating from individual vesicles and subsequently quantify the sample-induced Ca^2+^ influx using the following equation:

$$\text{Average }{\text{Ca}}^{2}+\text{ influx }=\;\left({\text{F}}_{\text{sample}}-{\text{F}}_{\text{blank}}\right)/\left({\text{F}}_{\text{ionomycin}}-{\text{F}}_{\text{blank}}\right)$$

All the imaging was done using a Total Internal Reflection Fluorescence Microscope (TIRFM) based on an inverted Nikon Ti microscope. For excitation, a 488 nm laser beam was focused in the back focal plane of the 60×, 1.49 NA oil-immersion objective lens. Fluorescence signal emerged from the samples collected from the same objective and imaged by air-cooled EMCCD camera. All the imaging experiments were performed at 293K. Fluorescence images were acquired using a 488 nm laser (∼10 W/cm^2^) for 50 frames with a scan speed of 20 Hz and analyzed using ImageJ to calculate the localized fluorescence intensity of each vesicle.

### Statistics

All statistical analyses were performed using Prism (GraphPad Software Inc). Nonparametric statistical tests were used when data did not show a normal distribution. The specific statistical test used in each experiment is described in the associated figure legend. Data are expressed as means ± SEM. Results were considered significant if *p* < 0.05. In most cases, the sample size (*i.e.*, the number of biological repeats, n) was 3, as indicated in the figure legends, and this number was selected as the default for experiments using isolated proteins, based on previous experience of variance sizes. In the case of Figure 3*I*, measuring the area of fibrils, the variance was found to be high (because the number of fibrils is low and size large), so n was increased to 8 for Aβ alone but remained at n = 3 for Aβ + WT or R47H sTREM2. For Figure 4*D*, which is a primary cell culture experiment, the sample size was 4, based on previous experience of variance sizes. The sample size n refers to biological repeats, which means repeating using a different biological sample preparation. Outliers were only encountered in the data of Figure 2*B* and were identified and removed by using GraphPad Prism outlier calculator.

## Data availability

The published article includes all data generated or analyzed during this study. Original source data for the figures in the paper are available upon request to the corresponding authors. No proprietary software was used in the data analysis. Further information and requests for resources and reagents should be directed to: gcb3@cam.ac.uk or phs22@cam.ac.uk

## Supporting information

This article contains supporting information.

## Conflict of interest

The authors declare that they have no conflicts of interest with the contents of this article.
