# Supplementary material for: Wild-type sTREM2 blocks Aβ aggregation and neurotoxicity, but the Alzheimer's R47H mutant increases Aβ aggregation
Source: J Biol Chem. 2021 Apr 3;296:100631. doi: 10.1016/j.jbc.2021.100631 (PMC8113883; doi:10.1016/j.jbc.2021.100631)
Supplement: Figures S1 to S15 and Table S1 [file mmc1.pdf]

Supporting Information

Wild-type sTREM2 blocks Aβ aggregation and neurotoxicity, but the Alzheimer's R47H mutant increases Aβ aggregation

Anna Vilalta[1†], Ye Zhou[2†], Jean Sevalle[2†], Jennifer K. Griffin[2†], Kanayo Satoh[2†], David H. Allendorf[1], Suman De[4], Mar Puigdellívol[1], Arturas Bruzas[1], Miguel A. Burguillos[1,3], Roger B. Dodd[3], Fusheng Chen[2], Yalun Zhang[2], Patrick Flagmeier[4], Lisa-Maria Needham[4], Masahiro Enomoto[5,] Seema Qamar[3], James Henderson[3], Jochen Walter[6], Paul E. Fraser[2], David Klenerman[4], Steven F. Lee[4], Peter St George-Hyslop[2,3*], Guy C. Brown[1*]

1 Table and 13 supplementary figures

Supplementary Table 1.

Dissociation constants, on and off rates for WT and R47H sTREM2 binding to Aβ oligomers. Fitted parameters for Supplementary Figures 6iii and iv.

	WT	R47H
**K_D1 (μM)**	2.00 ± 0.15	11.70 ± 5.97
**K_D2 (μM)**	0.29 ± 0.08	1.22 ± 0.30
**k_{on}1 (1/Ms)**	217300 ± 20200	59660 ± 3142
**k_{off}1 (1/s)**	0.4353 ± 0.0162	0.6986 ± 0.0304
**k_{on}2 (1/Ms)**	26250 ± 3054	6316 ± 342
**k_{off}2 (1/s)**	0.007717 ± 0.001845	0.007712 ± 0.001861
**R_{max}1**	0.6728	0.6637
**R_{max}2**	0.2158	0.1753
**Full R^2**	0.9951	0.9936

Supplementary Figure 1

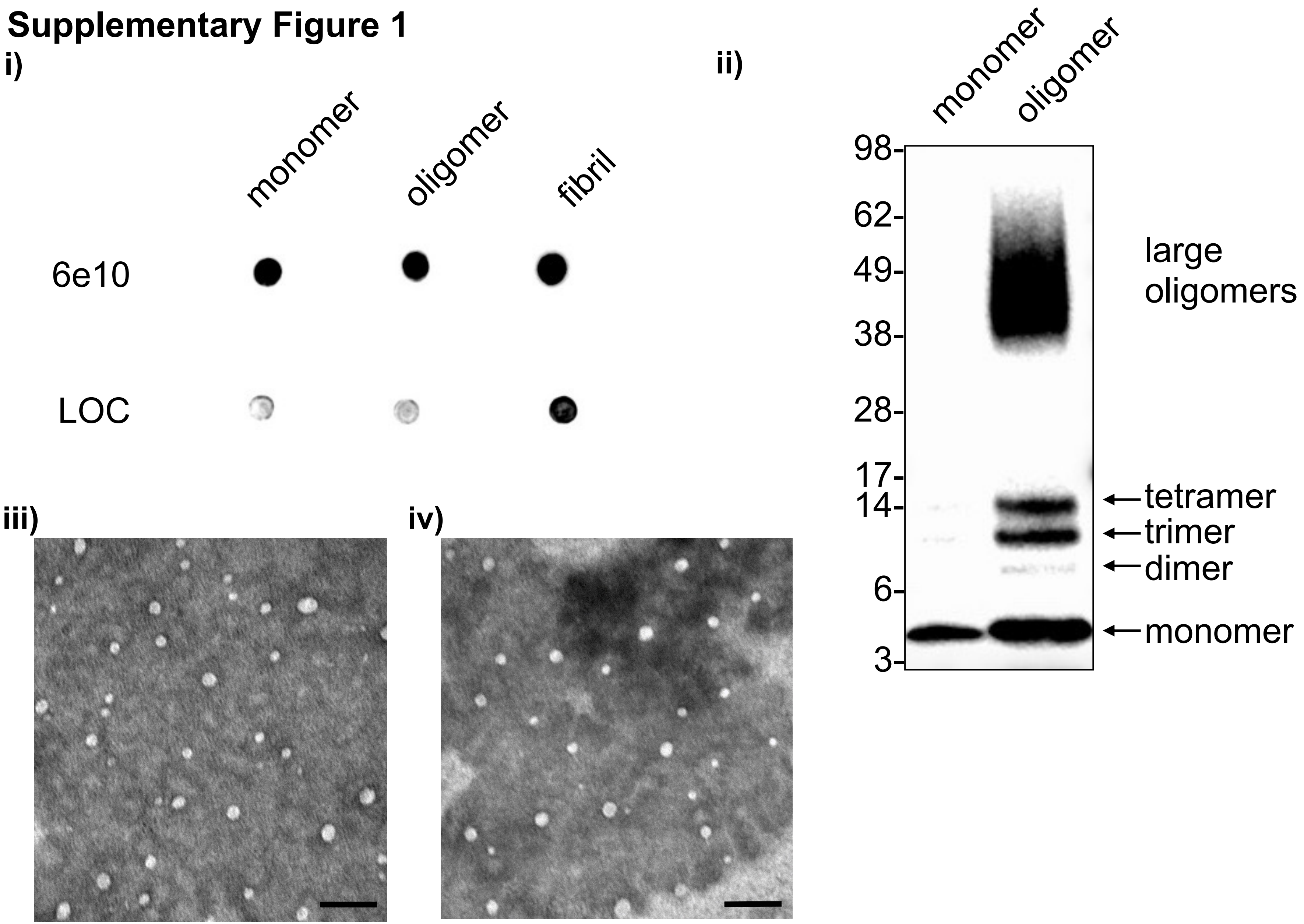

Supplementary Figure 1. Characterization of Aβ species,

Aβ42 monomers, oligomers and fibrils were prepared as previously described. i) Aβ monomers, oligomers and fibrils (20 ng of total Aβ) were applied to a nitrocellulose membrane and probed with mouse anti-Aβ antibody (6E10) or with rabbit anti-Amyloid fibrils antibody (LOC). ii) Aβ monomers and oligomers were identified by Western blot analysis. Aβ were separated by SDS-PAGE on a 4-12% NuPAGE bis-Tris gel and probed with anti-Aβ antibody (6E10). Note that less total Aβ protein was loaded in the monomer lane in order to not overload the monomer band. iii & iv) Characterization of Aβ species used in BLI experiments. Transmission electron microscopy (TEM) was performed using undiluted biotinylated Aβ (100%) or a mixture of biotinylated Aβ (10%) and unlabelled Aβ (90%) which were diluted to final concentrations of 1.1 μM. These TEM samples (10 μl) were applied to carbonate coated grids and negatively stained with 1% phosphotungstic acid (PTA). TEM micrographs indicating the globular morphology of the Aβ oligomers were obtained on a Hitachi H-7000 operated at 75kV. Scale bars are 100 nm.

Supplementary Figure 2

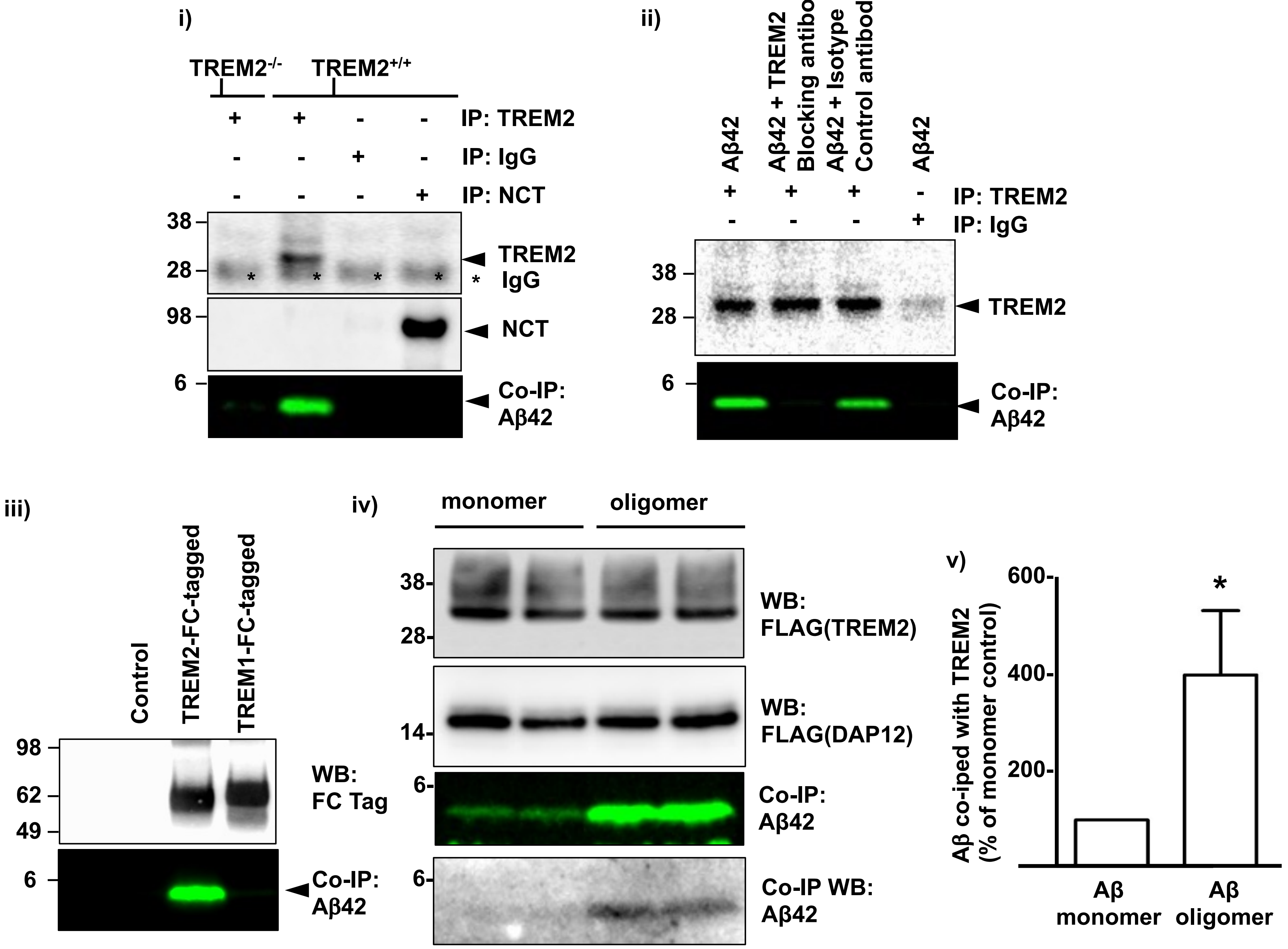

Supplementary Figure 2. Full-length TREM2 binds Aβ oligomers but not monomers.
i) Primary microglia were incubated with 100 nM HiLyte Fluor 647-labelled Aβ42 at 4°C for 1 hour, then lysed, immunoprecipitated with anti-TREM2 antibodies, and examined by SDS-PAGE and western blotting. Aβ oligomers co-immunoprecipitate with endogenous TREM2 in primary microglia (lane 2), but not with other natively endogenous Type I glycoproteins, such as nicastrin (lane 4) or with isotype control antibody (lane 3). TREM2 knockout microglia failed to co-immunoprecipitate Aβ (lane 1). **ii)** This interaction was blocked by preincubation with monoclonal anti-TREM2 blocking antibody (lane 2), but not by isotype control antibodies (lane 3). **iii)**: Aβ co-precipitated with recombinant FC-TREM2 (lane 2) but not FC-TREM1 (lane 3). **iv)** Aβ oligomers bind to TREM2 significantly better than monomers. HeLa cells transfected with DAP12-FLAG plus TREM2-FLAG were co-incubated with 100 nM freshly prepared monomeric or oligomeric HiLyte Fluor 647-labelled Aβ at 4°C for 1 hour. Prior to incubation, the concentration of monomer/oligomer were adjusted and tested according to fluorescence dot exposure to ensure total Aβ concentrations were the same in the working solutions of monomeric and oligomeric Aβ preparations. Cell membrane lysates were then immunoprecipitated with anti-hTREM2 antibody. The IP products were western blotted and probed with the indicated antibody. **v)** Aβ oligomers bind to TREM2 significantly better than monomers. Quantification of iv) with $n = 4$ replicates in 2 independent experiments; $p = 0.03$ Mann-Whitney U test). Error bars = SEM.

Supplementary Figure 3

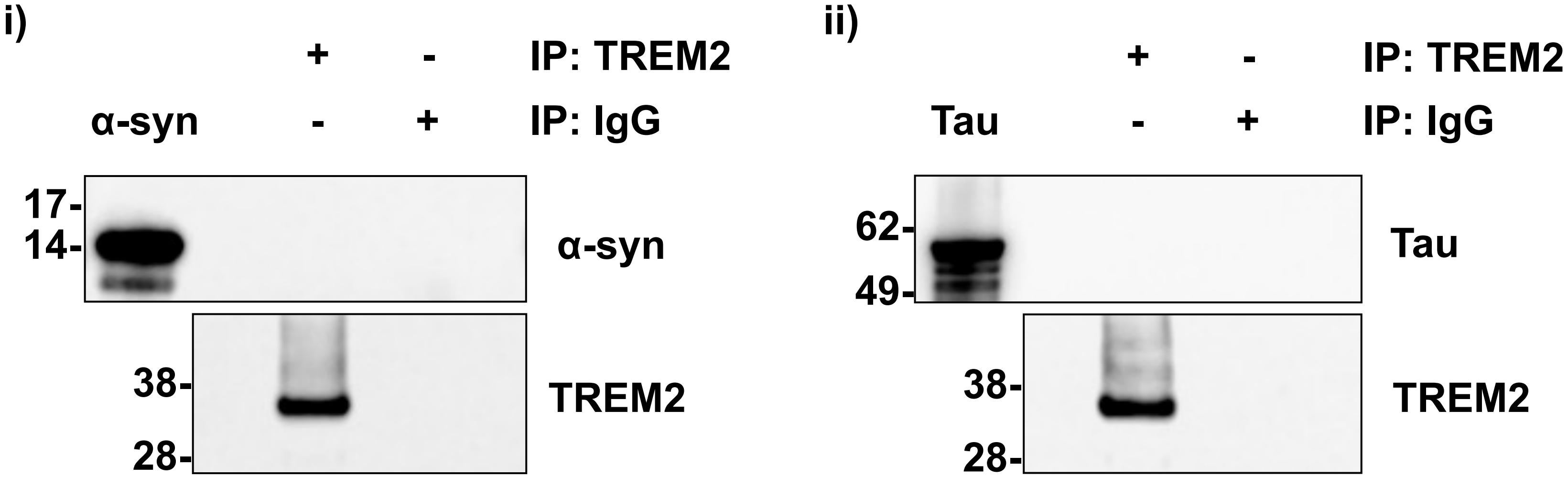

Supplementary Figure 3. TREM2 does not bind α-synuclein or Tau oligomers. Primary microglia were incubated with 100 nM oligomeric α-synuclein or Tau protein at 4°C for 1 hour. Cell membrane lysates were then immunoprecipitated with human TREM2 antibody or control IgG. The IP products were probed with the indicated antibody.

Supplementary Figure 4

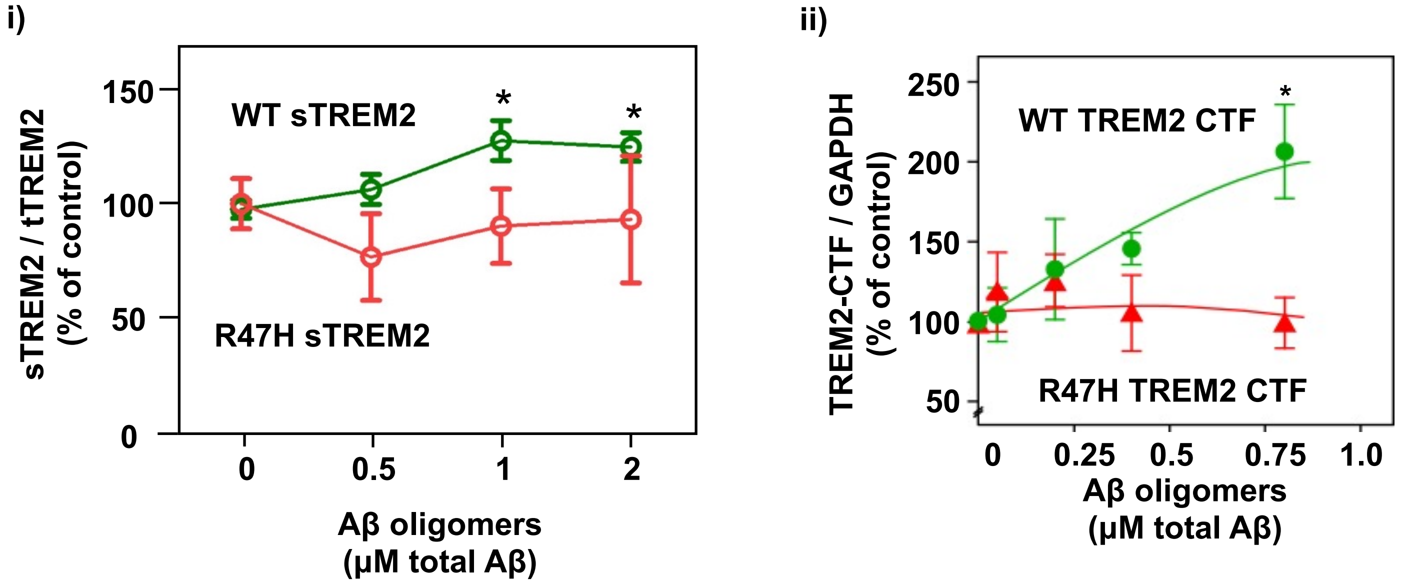

Supplementary Fig. 4. Aβ oligomers induce sTREM2 release and TREM2-CTF accumulation in cells expressing wild-type (but not R47H) TREM2. i) In primary murine microglia, endogenously expressing wild type TREM2 (WT-TREM2), addition of Aβ oligomers increased sTREM2 (normalised to total TREM2, green line). Aβ oligomers did not induce significant sTREM2 release in microglia from homozygous CRISPR-Cas9 engineered R47H TREM2 mice. Error bars = SEM; *= $p < 0.05$, n=4 independent experiments with ≥10 replications each; one-way ANOVA with Tukey's multiple comparisons post-test. **ii)** Addition of Aβ oligomers to HEK293 cells overexpressing wild-type TREM2 induced an increase in the membrane-bound C-terminal fragment of TREM2, measured 16 hours later, but did not do so in R47H TREM2 expressing cells (Supplementary Fig. 5).

Supplementary Figure 5

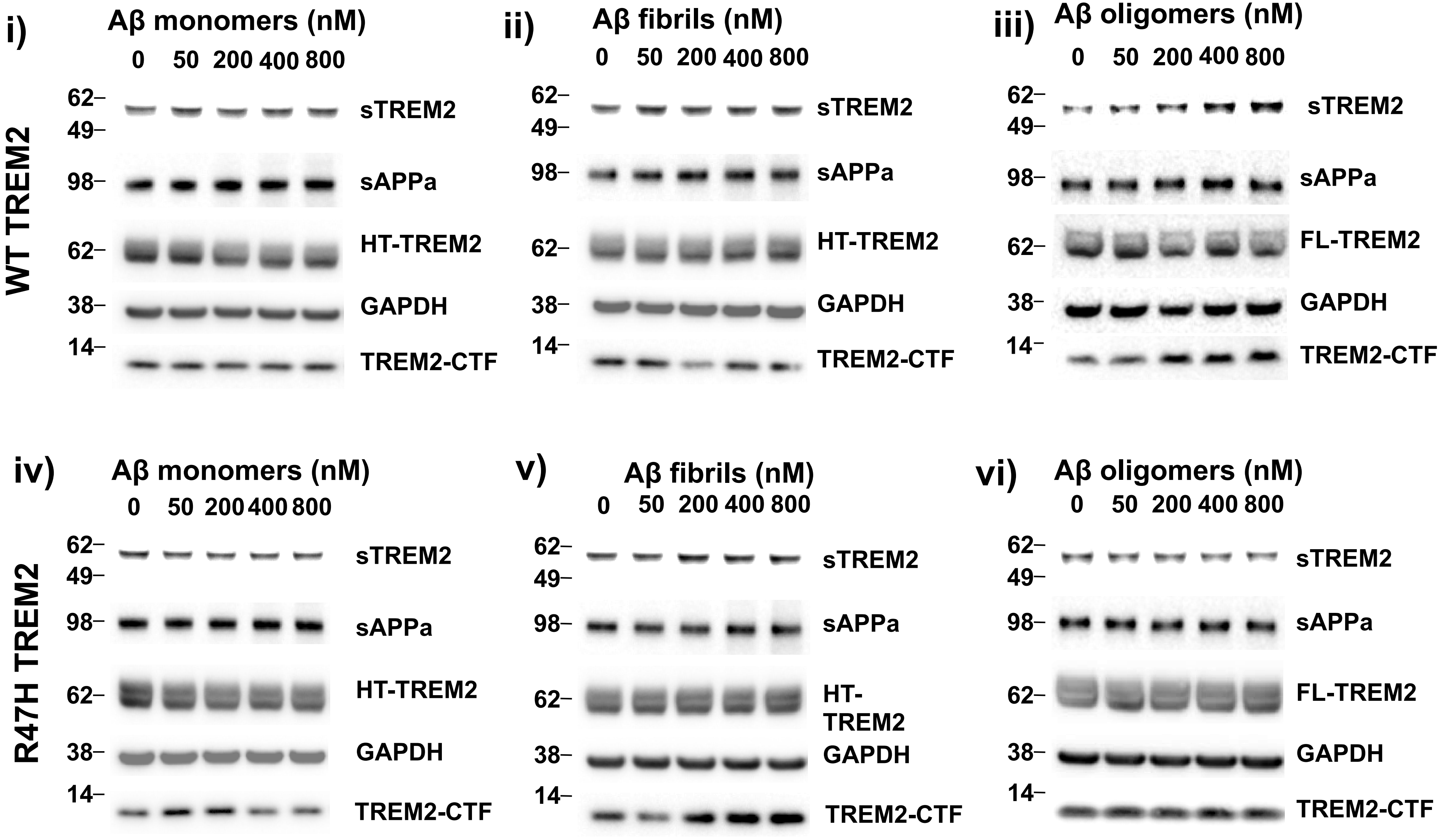

Supplementary Figure 5. Aβ monomers and fibrils fail to trigger TREM2 cleavage, but Aβ oligomers trigger TREM2 cleavage in wild-type but not R47H TREM2-expressing cells. Aβ monomers (i & iv) fibrils (ii & v) and oligomers (iii & vi) were added at the indicated concentrations to HEK293 cells co-expressing DAP12 and either wild-type (WT, I, ii & iii) or R47H TREM2 (iv, v & vi), both HaloTagged (HT-TREM2). TREM2 membrane-bound C-terminal fragment was assayed in cell lysates and sTREM2 from the culture medium 16 hours after Aβ addition. Representative western blots shown. Note that panel iii) is identical with Fig 1i of the main figures, but is included here to aid comparison with panels i and ii.

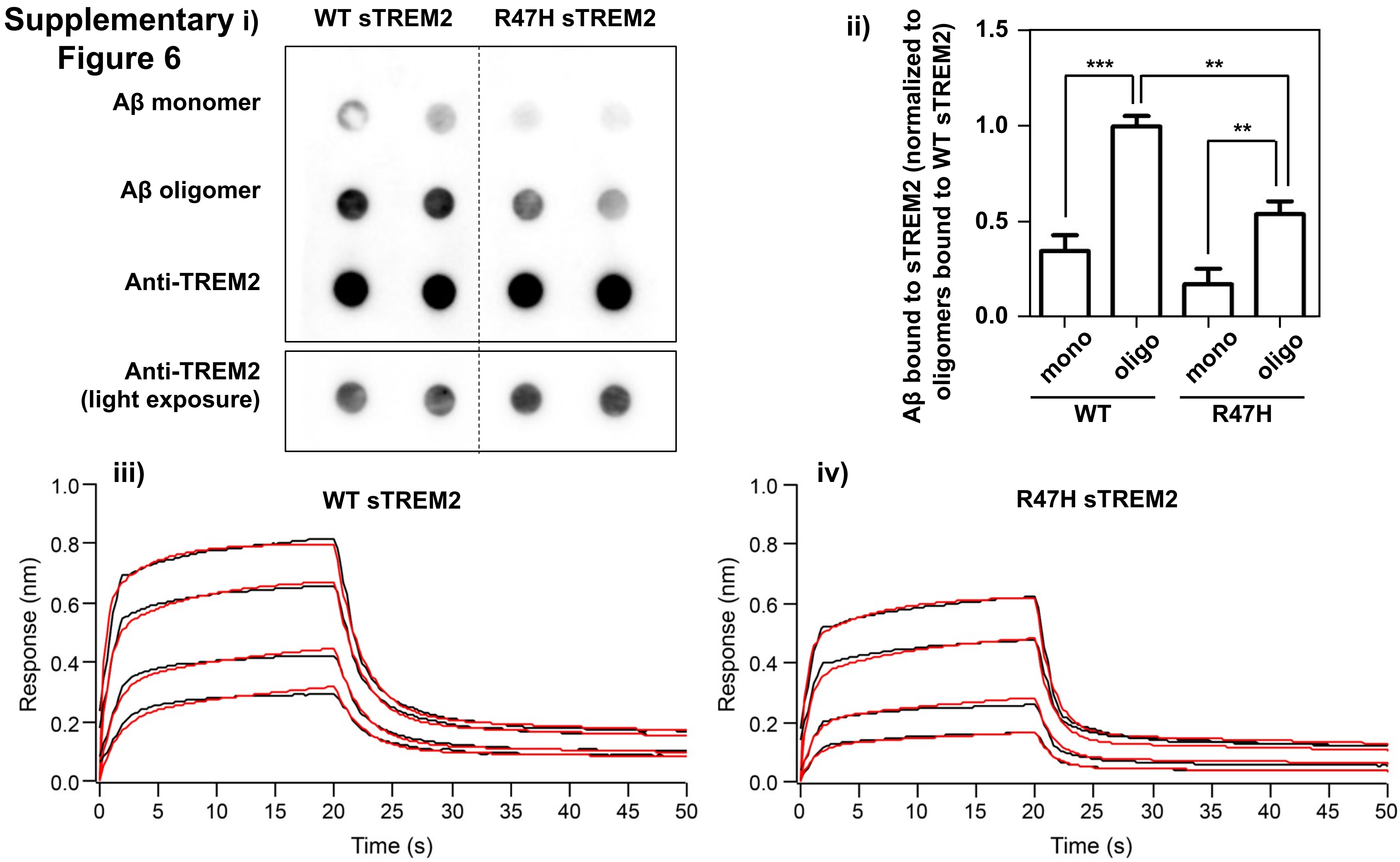

Supplementary Figure 6. Wild-type (WT) sTREM2 binds Aβ oligomers more than Aβ monomers, and R47H sTREM2 binds both less.
i) Dot Blot membrane strips spotted with Aβ monomers, oligomers and anti-TREM2 antibody were probed with WT sTREM2-His (left) or R47H sTREM2-His (right). Lower left panel is a lighter exposure of the anti-TREM2 antibody showing that similar amounts of sTREM2 were used.
ii) Quantification of sTREM2 bound to Aβ. Error bars = SEM; **=$p<0.01$, ***=$p<0.001$, n=3 independent experiments with 6 replications; one-way ANOVA with Tukey's post-hoc multiple comparisons test. **iii)** and **iv)** Bio-Layer Interferometry studies reveal a 2-state model for Aβ-binding to sTREM2. Curves of iii) WT sTREM2 or iv) R47H sTREM2 binding to Aβ oligomers at four different concentrations.. Black lines indicate experimental data. Red lines indicate fitted curves from the 2:1 model. The fitted parameters are shown in Table 1.

Supplementary Figure 7

i)

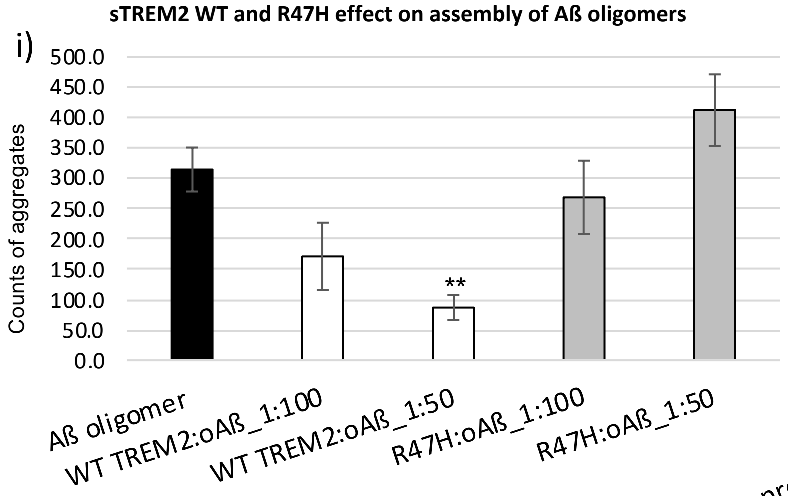

ii)

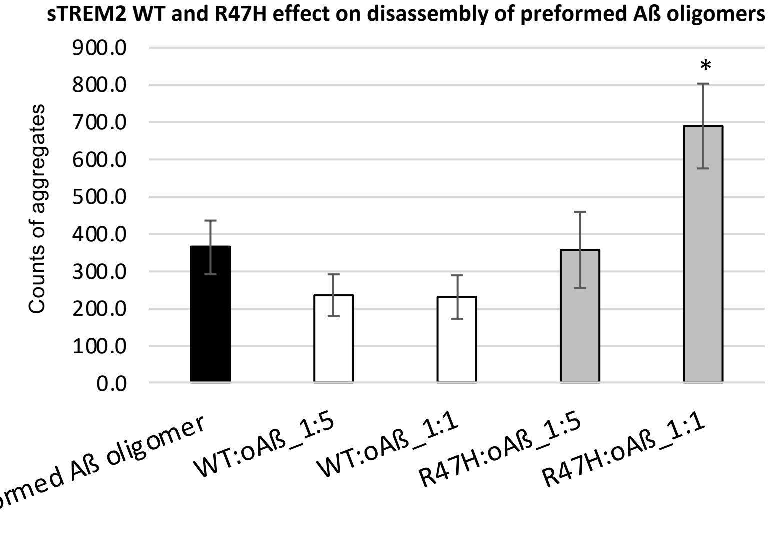

Supplementary Figure 7. WT sTREM2 decreases Aβ oligomer assembly, whereas R47H sTREM2 increases Aβ oligomer disassembly. i) Aβ was oligomerized in the presence of absence of WT or R47H sTREM2 (sTREM2 1:50 or 1:100 molar ratio to Aβ). After by transmission electron microscopy imaging (representative images in Fig 2), the numbers of Aβ aggregates (oligomers) were counted. **ii)** Aβ was oligomerized, and then incubated in the presence of absence of WT or R47H sTREM2 (sTREM2 1:5 or 1:1 molar ratio to Aβ). After imaging (representative images in Fig 2), the numbers of Aβ aggregates (oligomers) were counted. Error bars represent SD. Statistical analysis was performed using one-way ANOVA followed by Bonferroni's multiple comparison test (n=3, **p*<0.05, ***p*<0.01 vs Aβ oligomer alone).

Supplementary Figure 8

i) sTREM2 effects on Aβ oligomerization

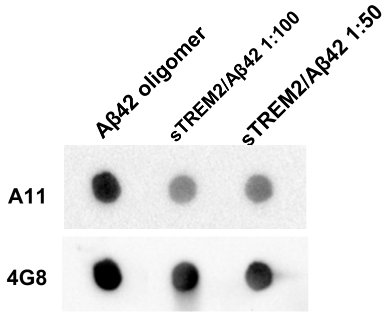

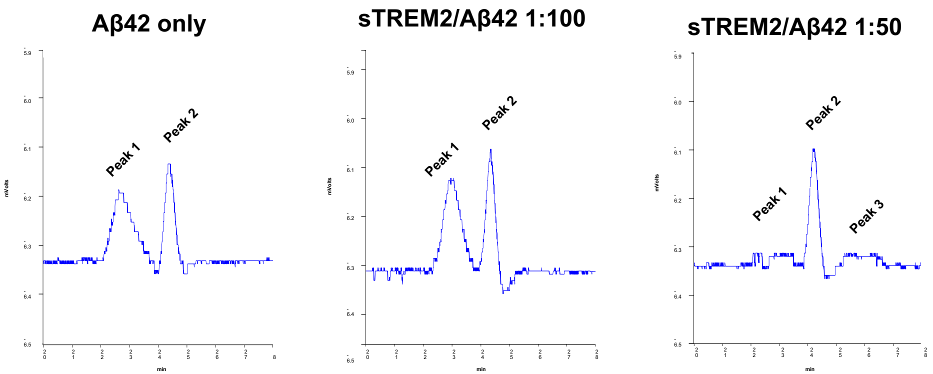

Samples name	Peak	peak area (mVoltage*sec)	peak width (sec)	retention time (min)
Aß42 only	Peak 1	8.01	44.4	22.61
	Peak 2	5.10	23.5	24.34
sTREM2/Aß42 1:100	Peak 1	7.80	41.1	22.94
	Peak 2	6.04	19.4	24.33
sTREM2/Aß42 1:50	Peak 1	1.15	51.2	22.81
	Peak 2	5.73	21.0	24.17
	Peak 3	3.07	70.2	25.30

ii) sTREM2 effects on reversal of Aβ oligomerization

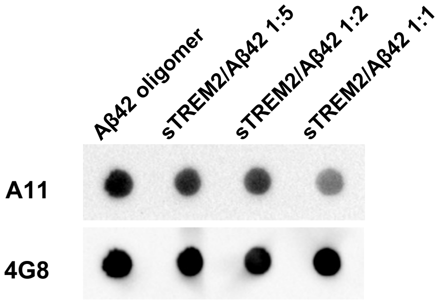

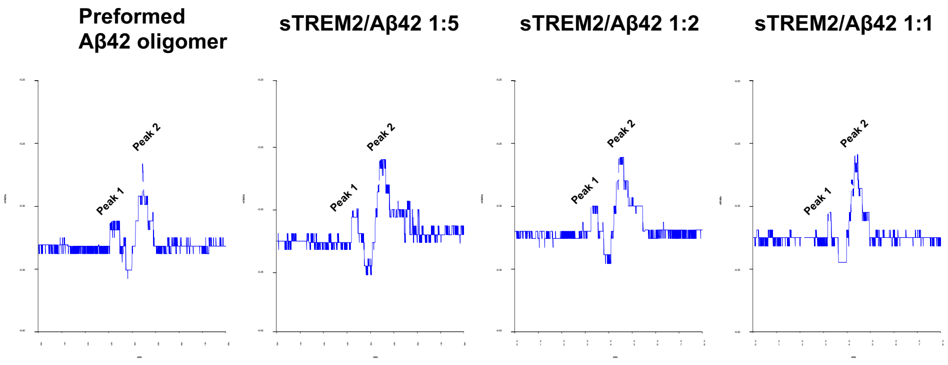

Samples name	Peak	peak area (mVoltage*sec)	peak width (sec)	retention time (min)
Preformed Aß42 oligomer	Peak 1	0.76	7.1	23.05
	Peak 2	1.94	4.5	24.43
sTREM2/Aß42 oligomer 1:5	Peak 1	0.746	16.6	23.23
	Peak 2	2.94	32.0	24.38
sTREM2/Aß42 oligomer 1:2	Peak 1	0.60	18.2	23.24
	Peak 2	3.53	30.8	24.42
sTREM2/Aß42 oligomer 1:1	Peak 1	0.417	9.0	23.18
	Peak 2	2.31	28.5	24.32

Supplementary Figure 8. Wild-type sTREM2 inhibits oligomerisation of monomeric Aβ42, and disaggregates preformed Aβ oligomers. The same Aβ preparations examined in Figure 2i by transmission electron microscopy were also investigated by HPLC-SEC (left panels and table). In both i) Aβ oligomer formation and ii) Aβ oligomer disaggregation experiments, sTREM2 reduced the area under "Peak 1", and increased the area under "Peak 2" and in some instances caused the appearance of a "Peak 3". The same Aβ preparations were also examined by dot blot hybridisation against the A11 anti-oligomer antibody and the 4G8 anti-Aβ antibody (right panels). In good agreement with both the TEM and the HPLC-SEC studies, the presence of sTREM2 was associated with reductions in A11 immunoreactive oligomeric Aβ species.

Supplementary Figure 9

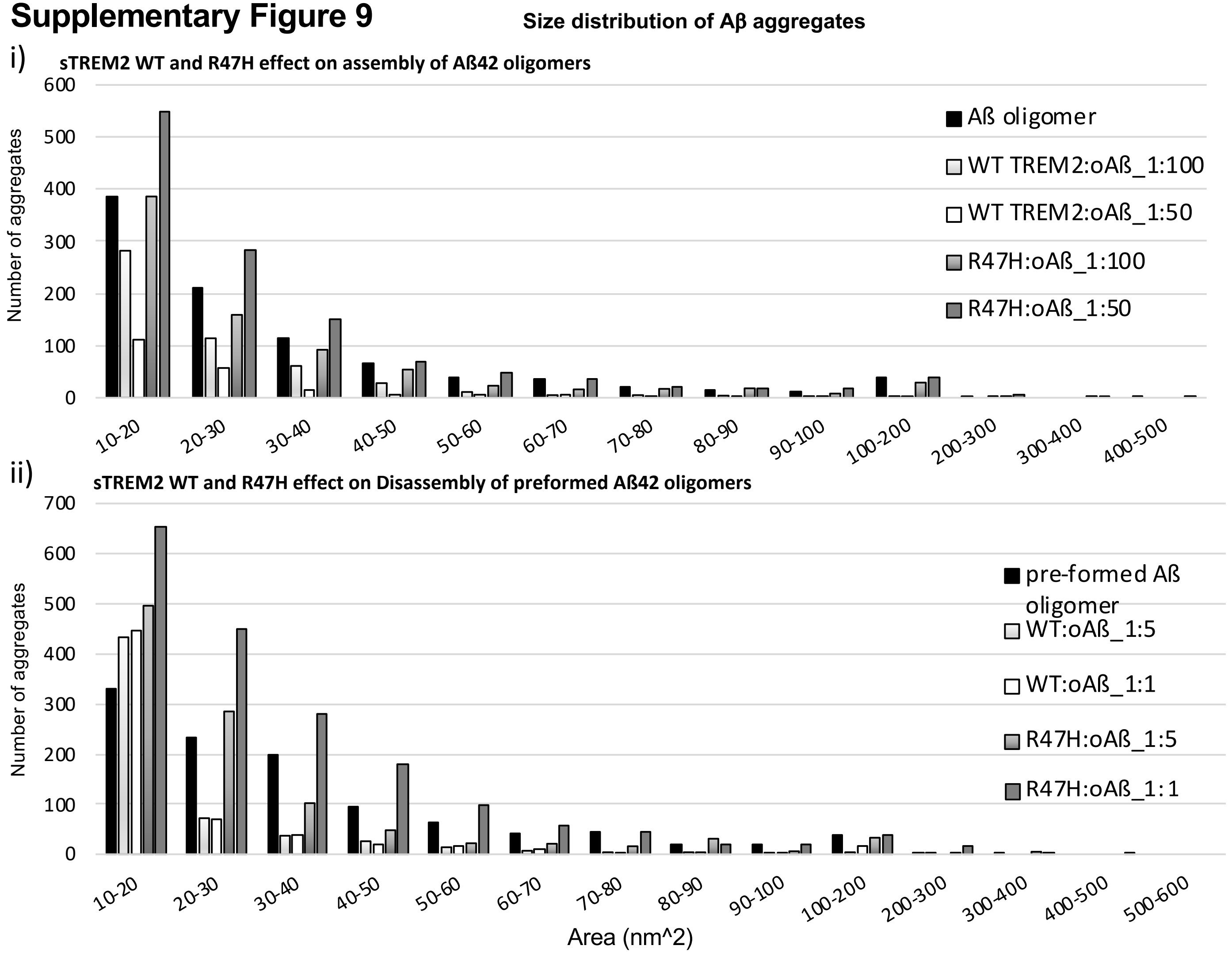

Supplementary Figure 9. WT sTREM2 preferentially inhibits the production of large Aβ oligomers, and disaggregates large Aβ oligomers into small oligomers; R47H sTREM2 increases the number of small Aβ oligomers. i) Quantification of the size distribution of Aβ aggregates (oligomers) produced by oligomerizing Aβ in the presence or absence of WT or R47H sTREM2 at the indicated molar ratios. **ii)** Quantification of the size distribution of Aβ aggregates (oligomers) resulting from incubation of preformed Aβ oligomers ± WT or R47H sTREM2 at the indicated molar ratios. Representative TEM images in Fig 2. Data from three independent TEM images.

Supplementary Figure 10

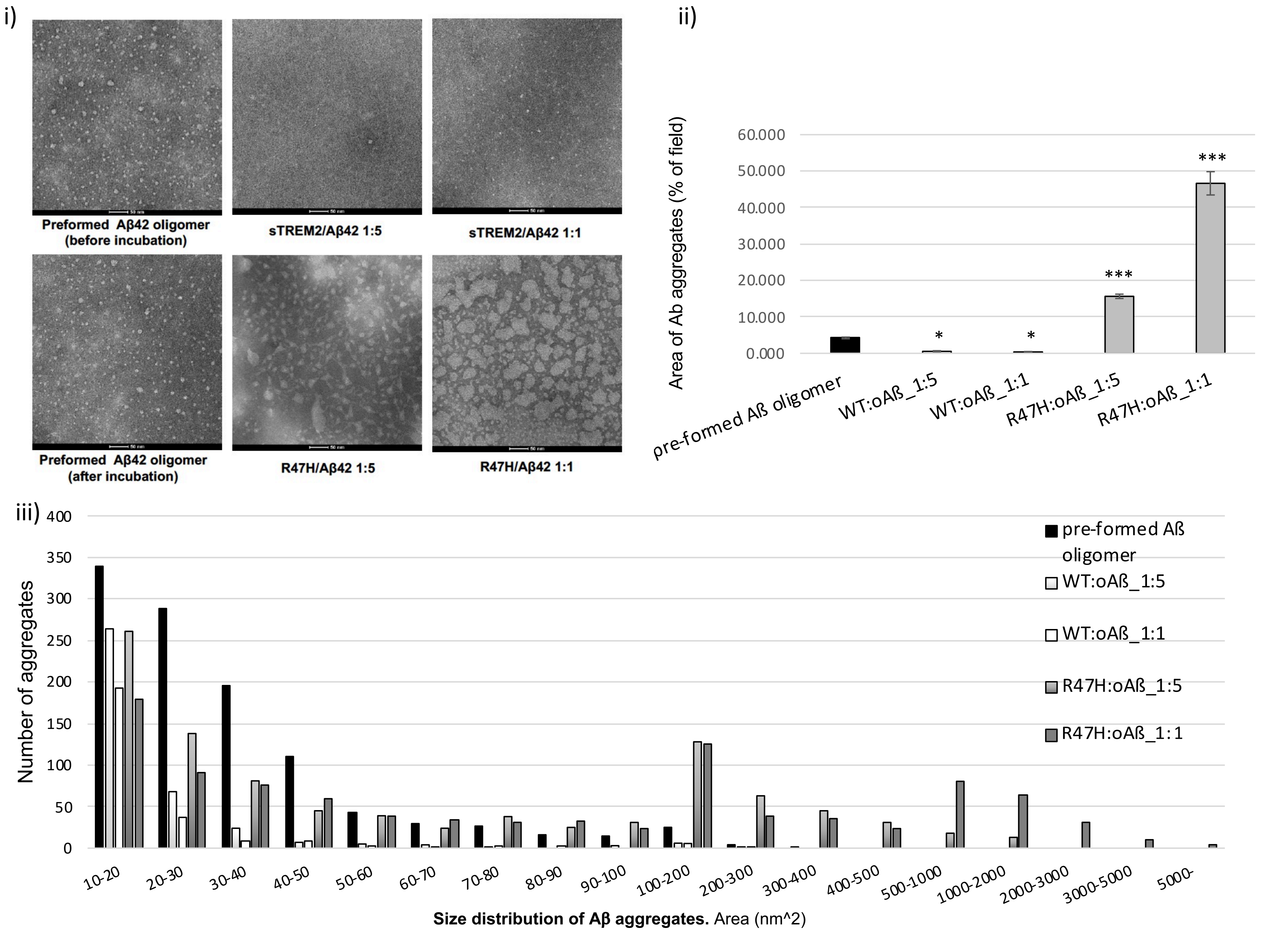

Supplementary Figure 10. At low concentrations of Aβ, wild-type sTREM2 dissolves Aβ oligomers, whereas R47H sTREM2 induces very large Aβ aggregates: total area and size distribution of Aβ aggregates. Preformed Aβ oligomers were incubated at 100 nM Aβ (monomer equivalent) ± 20 or 100 nM wild-type or R47H sTREM2 for 30 mins at 37°C, then TEM imaged. i) Representative TEM images. ii) Quantification of total area of Aβ aggregates. Error bars represent SD. Statistical analysis was performed using one-way ANOVA followed by Bonferroni's multiple comparison test (n=4, *p<0.05, ***p<0.001 Aβ oligomer alone). iii) Size distribution of the aggregates on from three independent TEM images.

Supplementary Figure 11

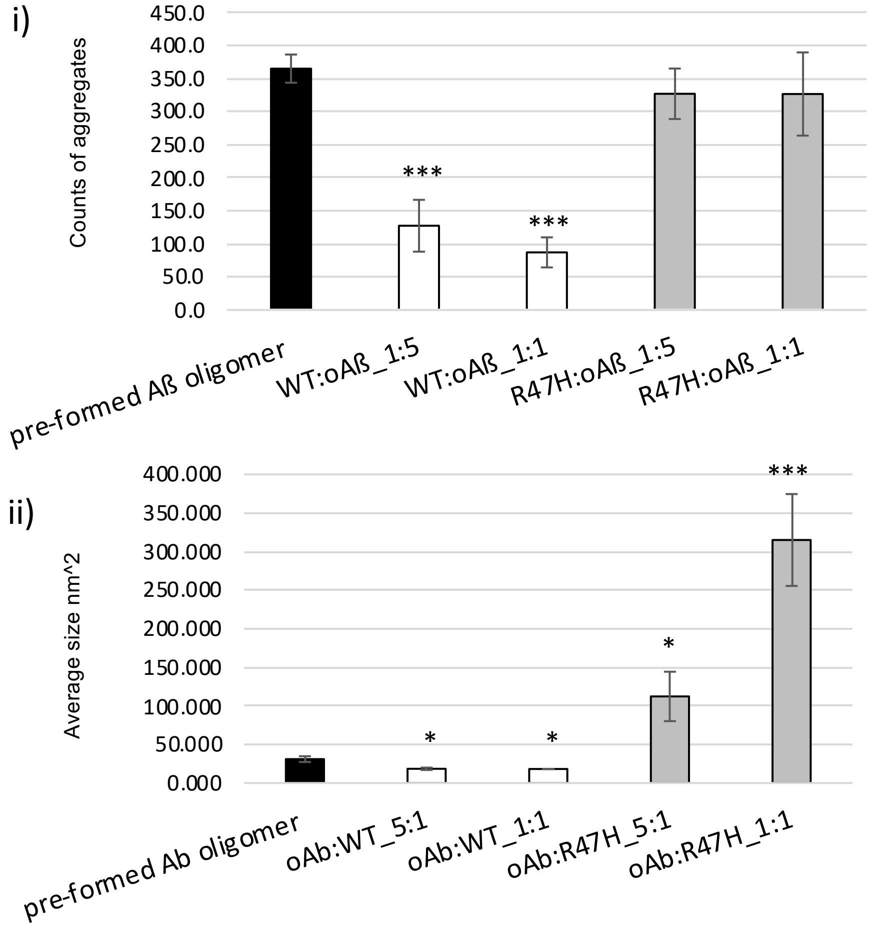

Supplementary Figure 11. At low concentrations of Aβ, wild-type sTREM2 dissolves Aβ oligomers, whereas R47H sTREM2 induces very large Aβ aggregates: numbers and sizes of Aβ aggregates. Further analysis of the aggregates of Supplementary Fig. 10. Preformed Aβ oligomers were incubated at 100 nM Aβ (monomer equivalent) ± 20 or 100 nM wild-type or R47H sTREM2 for 30 mins, then TEM imaged. i) Number of Aβ aggregates. Ii) Average size (area) of each Aβ aggregate. Error bars represent SD. Statistical analysis was performed using one-way ANOVA followed by Bonferroni's multiple comparison test (n=4, **p*<0.05, ****p*<0.001 Aβ oligomer alone).

Supplementary Figure 12

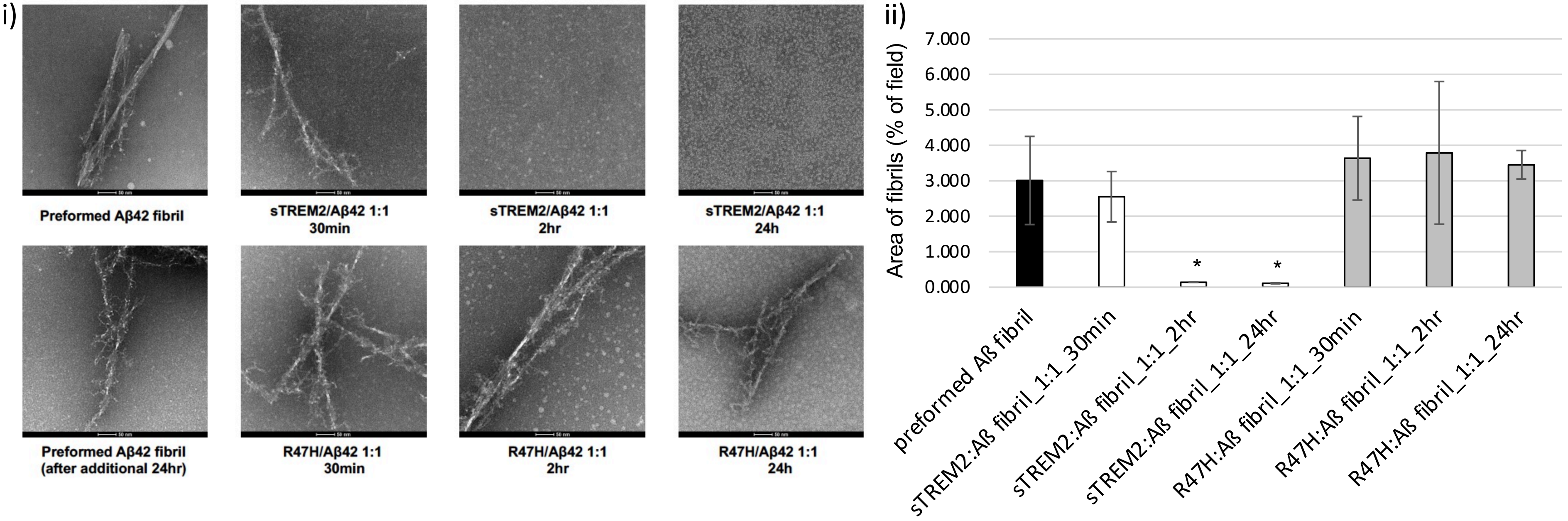

Supplementary Figure 12. WT sTREM2 disaggregates preformed Aβ fibrils. Time course for experiment depicted in Fig. 3. **i)** Preformed Aβ fibrils were treated ± WT or R47H sTREM2 (at a 1:1 molar ratio) for 30min, 2hrs or 24 hrs. Negative-stain TEM revealed that WT sTREM2 dissociated Aβ fibrils after 2 hour incubation but R47H did not. **ii)** Quantification of the area of Aβ fibrils. Some of the images and data here are reproduced in Fig 3. Error bars represent SD. Statistical analysis was performed using one-way ANOVA followed by Bonferroni's multiple comparison test (n=3-8,*$p<0.05$ vs pre-formed Aβ fibril).

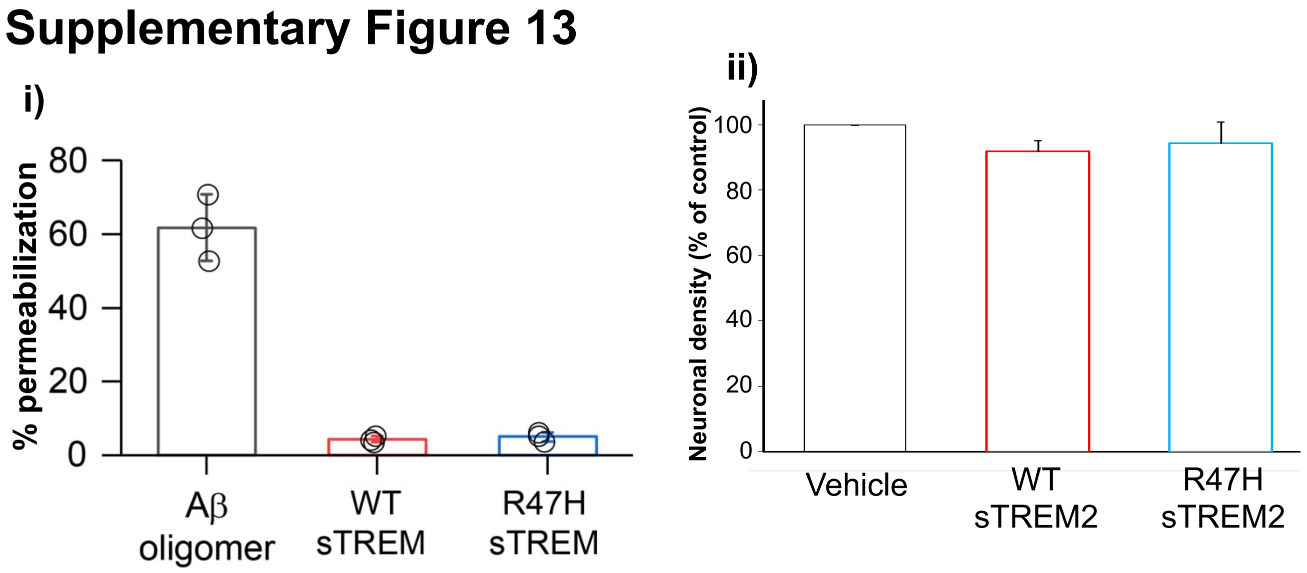

Supplementary Figure 13. In the absence of Aβ, wild-type and R47H sTREM2 have no effect on i) membrane permeability or ii) neuronal density. **i)** 1 µM Aβ, WT sTREM2 or R47H sTREM2 were incubated (separately) for 6 hours and diluted to 200 nM before the membrane-permeabilization assay was performed. Error bars = SEM; n=3 independent experiments. ii) Mixed neuronal-glial co-cultures were treated with either: vehicle, 40 nM wild-type sTREM2 or 40 nM R47H sTREM2, and 3 days later neuronal density was counted. Error bars = SEM; n=4 independent experiments on different cell cultures.

Supplementary Figure 14

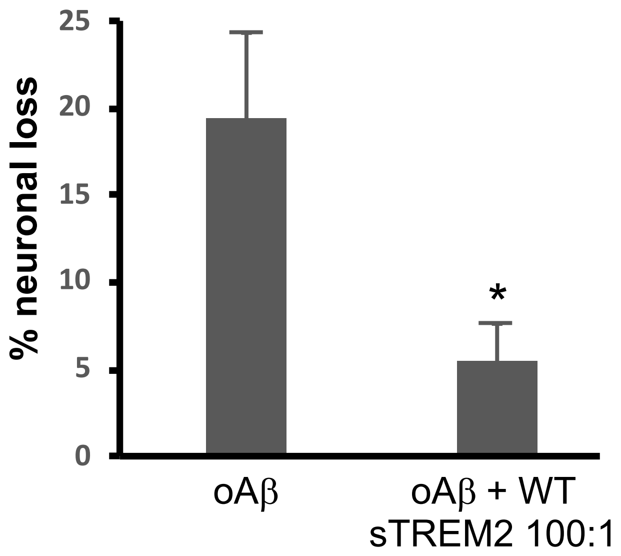

Supplementary Figure 14. Wild-type sTREM2 reduced the neuronal loss induced by oligomeric Aβ. Mixed neuronal-glial co-cultures were treated with either: vehicle, 4 μM oligomeric Aβ or 4 μM oligomeric Aβ plus 40 nM wild-type sTREM2, and 3 days later neuronal density was counted. Neuronal loss is the % loss of neurons in wells treated with Aβ relative to wells treated with vehicle. The number of dead neurons was not significantly different between treatments. Error bars = SEM; n=4 independent experiments on different cell cultures. Significance was tested by unpaired t-test. * $p<0.05$.

Supplementary Figure 15

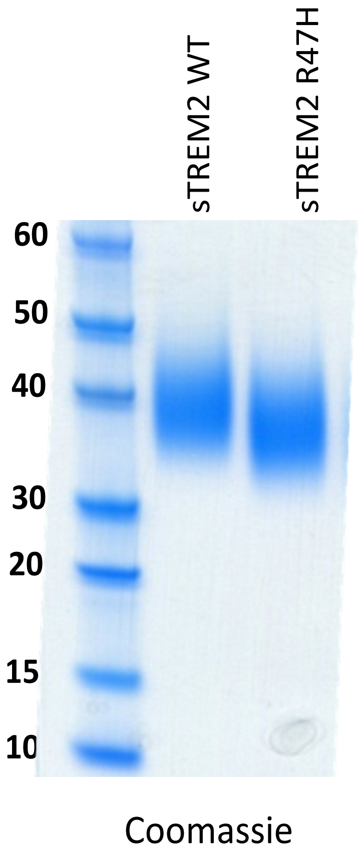

Supplementary Figure 15. Purified wild-type and R47H sTREM2 are indeed pure. Purified WT and R47H sTREM2 were run on SDS-PAGE gels, with molecular weight markers, and then the gel was stained with Coomassie to reveal all proteins.
